# The time-effect relationship of intra-articular ozone injection for knee osteoarthritis: a systematic review and meta-analysis

**DOI:** 10.3389/fpain.2026.1843756

**Published:** 2026-06-17

**Authors:** Fengjiao Chen, Yufeng Tao, Jing Deng, Leyi Zhang, Lanlan Yu, Zhuoxi Yang, Yixuan Zhang, Chi Zhang

**Affiliations:** 1 School of Health Preservation and Rehabilitation, Chengdu University of Traditional Chinese Medicine, Chengdu, Sichuan, China; 2 School of Clinical Medicine, Chengdu University of Traditional Chinese Medicine, Chengdu, Sichuan, China

**Keywords:** knee osteoarthritis, meta-analysis, ozone therapy, systematic review, time-course

## Abstract

**Background:**

As a gradual degenerative illness, knee osteoarthritis (KOA) is characterized by high rates of impairment and prevalence. Patients’ quality of life is seriously jeopardized, and healthcare systems and social advancement are continuously burdened. Because it is minimally invasive, intra-articular injection treatment is frequently used to treat KOA. Among these, ozone therapy has drawn more and more clinical interest because of its ease of use, affordability, and observable symptom relief. Its scientific application in long-term management strategies is limited, nonetheless, because the exact length of its therapeutic benefits is still unknown.

**Objective:**

This study is to integrate clinical data from various follow-up time points using systematic review and meta-analysis techniques in order to comprehensively assess the temporal efficacy of intra-articular ozone therapy in reducing symptoms among patients with KOA. In order to provide evidence-based support and decision-making references for phased and continuous clinical treatment planning, the goal is to elucidate the temporal aspects of persistent therapeutic effects.

**Methods:**

Searches were conducted in PubMed, Embase, the Cochrane Library, Web of Science, and Scopus from the inception of each database up to December 3, 2025. Two reviewers independently extracted data using the Cochrane Risk of Bias Assessment Tool and the Grading of Recommendations, Assessment, Development and Evaluation (GRADE) approach, and assessed the quality of the evidence and the certainty of the evidence for each outcome measure. Outcomes included Western Ontario and McMaster Universities Osteoarthritis Index (WOMAC) pain, WOMAC stiffness, WOMAC physical function, WOMAC Total, and Visual Analog Scale (VAS) pain. For combined outcomes, standardized mean difference (SMD) and 95% confidence interval (CI) were calculated. Statistical analysis and graphing of included information were performed using Review Manager 5.4.1, Stata 15.0, and GRADEpro GDT.

**Results:**

A total of 16 randomized controlled trial (RCT) studies (*n* = 1,172) were included in this review. Meta-analysis results demonstrated that intra-articular ozone therapy exhibits a clear time-dependent effect on symptom improvement in patients with KOA. At short-term follow-up, the WOMAC total decreased at 1 month post-intervention [SMD = − 0.63, 95% CI (−1.17, −0.10), *P* < 0.01]; at 2 months, WOMAC pain[SMD = − 1.39, 95% CI (−1.96, −0.81), *P* < 0.01], WOMAC function [SMD = − 1.27, 95% CI (−2.06, −0.49), *P* < 0.01], and VAS[SMD = − 1.25, 95% CI (−2.10, −0.40), *P* < 0.01] all showed significant improvement. However, During the 3-month follow-up, no statistically significant differences were observed in any of the outcomes. At 6 and 12 months, compared with the control group, the ozone group showed statistically significantly worse scores in several outcomes, suggesting that ozone not only lacks sustained efficacy but may also be inferior to active control therapies in the long term.

**Conclusions:**

Intra-articular ozone therapies are effective in alleviating pain and improving joint function in patients with knee osteoarthritis in the short term (1–2 months), with effects peaking at 2 months. By 3 months, the therapeutic effect diminishes, and by 6 or 12 months, the evidence of efficacy is very weak, whereas active comparators (such as hyaluronic acid) demonstrate superior therapeutic outcomes at these time points. These findings suggest that ozone therapy may serve as a short-term treatment option but also underscore the need for placebo-controlled long-term trials and studies on repeated dosing regimens before recommending repeat injections.

**Systematic Review Registration:**

identifier CRD420251169941.

## 1 Introduction

Knee osteoarthritis (KOA) is a chronic degenerative joint disease with high global prevalence and disability rates. It is a primary cause of pain and functional impairment in middle-aged and elderly individuals, imposing a significant burden on patients’ quality of life, healthcare systems, and socioeconomic outcomes (1). Among numerous treatment strategies, conservative therapies focused on symptom relief and functional improvement hold significant importance (2, 3). Intra-articular injection therapy, characterized by its minimally invasive nature, relative safety, and direct targeting of affected sites, has gained widespread clinical application (4).

Ozone therapy, as a form of intra-articular injection, has garnered significant attention in recent years due to its simplicity, low cost, and favorable short-term safety profile. Existing randomized controlled trials (RCTs) and previous meta-analyses have confirmed that intra-articular ozone therapy effectively alleviates pain and improves function in patients with knee osteoarthritis (5). However, systematic evidence regarding the durability of its therapeutic effects remains limited. To date, only two relevant meta-analyses exist (6, 7), both published in 2019. These studies are dated, and their conclusions are limited by the small number of included trials and the sparse distribution of follow-up time points. These limitations create critical gaps in understanding the dynamic evolution of treatment effects and leave two core clinical questions unanswered: First, how does the precise efficacy of ozone therapy change across different stages? Second, at what point does its symptom-relieving effect begin to show statistically and clinically significant attenuation? Clarifying these temporal characteristics is crucial for establishing scientifically grounded treatment intervals (determining when repeat injections are necessary) and planning long-term management strategies.

To address this evidence gap, this study aims to integrate the latest clinical research evidence through systematic review and meta-analysis methods. For the first time, it conducts stratified and comparative analyses based on five predefined intensive follow-up time points (1, 2, 3, 6, and 12 months). The primary objective of this study is to precisely evaluate the differences in the efficacy of intra-articular ozone injection on knee osteoarthritis outcome measures at various time points, thereby providing direct evidence for establishing evidence-based, individualized treatment and re-intervention protocols in clinical practice.

## 2 Methods

This systematic review and meta-analysis strictly adhered to the Preferred Reporting Items for Systematic Reviews and Meta-Analyses (PRISMA) guidelines (8). The review team conducted the systematic review and meta-analysis according to the PRISMA checklist (Supplementary Material Table S1. PRISMA). This protocol was registered in the Prospective Register of Systematic Reviews (http://www.crd.york.ac.uk/PROSPERO, ID: CRD420251169941) before the review was conducted.

This review was first submitted to PROSPERO on October 16, 2025 (Version 1.0). Following an initial literature search and data extraction, the protocol was revised, and the revised version (Version 2.0) was published on December 2, 2025 (see the PROSPERO revision note). The final literature search was conducted on December 3, 2025, at which point the protocol revision was complete. All analyses reported in this manuscript were conducted in accordance with the revised protocol.

### 2.1 Search strategy

Two independent reviewers (FC and YT) conducted the literature search across five databases: PubMed, Embase, Cochrane Library, Web of Science, and Scopus. We included articles published in English and Chinese from the inception of each database up to December 3, 2025. Medical Subject Headings (MeSH) terms included “knee osteoarthritis,” “ozone therapy,” “ozone,” “randomized controlled trial,” their synonyms, and combinations thereof. A detailed search strategy is provided in the supplementary materials (Supplementary Material Table S2. Search strategy).

### 2.2 Inclusion and exclusion criteria

The inclusion criteria for this study were: (1) The study design was a RCT; (2) All subjects were diagnosed with KOA using authoritative diagnostic systems such as American College of Rheumatology classification criteria (ACR) and the Kellgren Lawrence classification (KL), based on imaging evidence or physician confirmation, without restrictions on gender, age, ethnicity, disease duration, or severity; (3) Assessment of the following outcome measures: Western Ontario and McMaster Universities Osteoarthritis Index (WOMAC) Total, WOMAC pain, WOMAC stiffness, WOMAC physical function, and Visual Analogue Scale (VAS) pain; (4) The intervention group received intra-articular ozone therapy without restrictions on concentration, dosage, or frequency. The control group received unrestricted interventions, which could include placebo, hyaluronic acid, or other treatments.

Exclusion criteria were: (1) not RCT; (2) absence of original data or incomplete data; (3) inability to obtain full-text articles; (4) non-compliance with intervention protocols in the intervention group, such as combining injections with other medications or administering injections both intra-articularly and periarticularly.

### 2.3 Research selection

All retrieved articles were imported into EndNote 21 reference management software. Initial duplication checks were performed based on article titles and DOIs, with duplicate articles excluded. Subsequently, two reviewers (FC and JD) independently screened the titles and abstracts of the remaining articles against predetermined inclusion and exclusion criteria, excluding studies that were clearly irrelevant. For studies preliminarily meeting the criteria, full texts were obtained and read to assess their final eligibility. Throughout the screening process, any disagreements between the two reviewers were resolved through consultation with a third reviewer (LZ).

### 2.4 Data extraction

Two reviewers (FC and YT) independently extracted key information from the final selected articles and entered it into Microsoft Excel 2021. Key information extracted included: first author, publication year, country, participant age, diagnostic criteria, sample size, intervention measures (intervention and control groups), concentration, dosage, and frequency of the intervention group, outcome measures (WOMAC/VAS), and adverse reactions. In cases of missing information, we emailed the first author for clarification. If no response was received after two inquiries, the study was excluded. After completing all data extraction, two reviewers cross-checked the information. Discrepancies between reviewers were resolved through discussion. If unresolved, a third reviewer (JD) provided recommendations to ensure data accuracy.

### 2.5 Risk of bias assessment and GRADE

The assessment was conducted independently by two reviewers (FC and JD). In case of disagreement, a third reviewer (LZ) resolved the issue through consultation. Risk of bias assessment for included studies utilized the Cochrane Risk of Bias Assessment Tool Version 1.0 (9). Each domain was rated as “low risk of bias”, “high risk of bias” or “Unclear risk of bias” according to the tool's guidelines. The tool assessed the following aspects: random sequence generation, allocation concealment, blinding of investigators and participants, blinding of outcome assessors, completeness of outcome data, selective reporting of results, and other potential sources of bias. The quality of evidence for outcomes measured at different time points was graded into four levels: high, moderate, low, and very low. Following GRADE criteria, evidence downgrading is considered for five factors: risk of bias, inconsistency, indirectness, imprecision, and publication bias.

### 2.6 Assessment of reporting quality

To ensure the standardization, transparency, and completeness of this systematic review report process, the report quality of this study adopts PRISMA 2020 statement as the evaluation standard. This statement comprises 27 items covering sections including title, abstract, introduction, methods, results, discussion, and funding. Each item assesses the adequacy of reporting for its corresponding content. Evaluation was conducted by two reviewers (FC and YT). Any discrepancies were resolved through consultation with a third reviewer (JD).

### 2.7 Statistical analysis

This study employed Review Manager 5.4.1 statistical software to conduct research quality assessments, data pooling, heterogeneity testing, and forest plot generation for the included studies. The primary outcome measures comprised the WOMAC Total, WOMAC Pain, WOMAC Stiffness, WOMAC physical function, and VAS pain. Quantitative data were extracted from all selected RCTs, including sample sizes and mean ± standard deviation (SD) for baseline and post-intervention measurements in each group. Raw data from all RCTs and calculations of means and SDs are presented in the supplementary material (Supplementary Material Table S3). Given that all variables included in the studies were reported as continuous data, we used standardized mean difference (SMD) with 95% confidence interval (CI) to estimate effect sizes. Clinically, effect sizes based on SMD were categorized as small (<0.40), moderate (0.40–0.70), or large (>0.70) (10). We considered *p* < 0.05 as statistically significant.

Assessing heterogeneity among studies using the *I*^2^ value: *I*^2^ ≤ 25% indicates low heterogeneity; 25% < *I*^2^ < 50% indicates moderate heterogeneity; 50% < *I*^2^ < 75% indicates high heterogeneity; *I*^2^ ≥ 75% indicates very high heterogeneity (11). When *I*^2^ < 50% and *p* ≥ 0.1, a fixed-effects model was used for meta-analysis; otherwise, a random-effects model was employed. For studies with high heterogeneity, subgroup analysis and sensitivity analysis were conducted using Review Manager 5.4.1 and Stata 15.0 software to assess the stability of meta-analysis results.

To examine the temporal dynamics of treatment efficacy, the core analysis will be conducted at different follow-up time points (e.g., 1 month, 2 months, 3 months, 6 months, 12 months), comparing the differences in effect sizes across these time points.

## 3 Results

### 3.1 Search results

**Figure 1:**
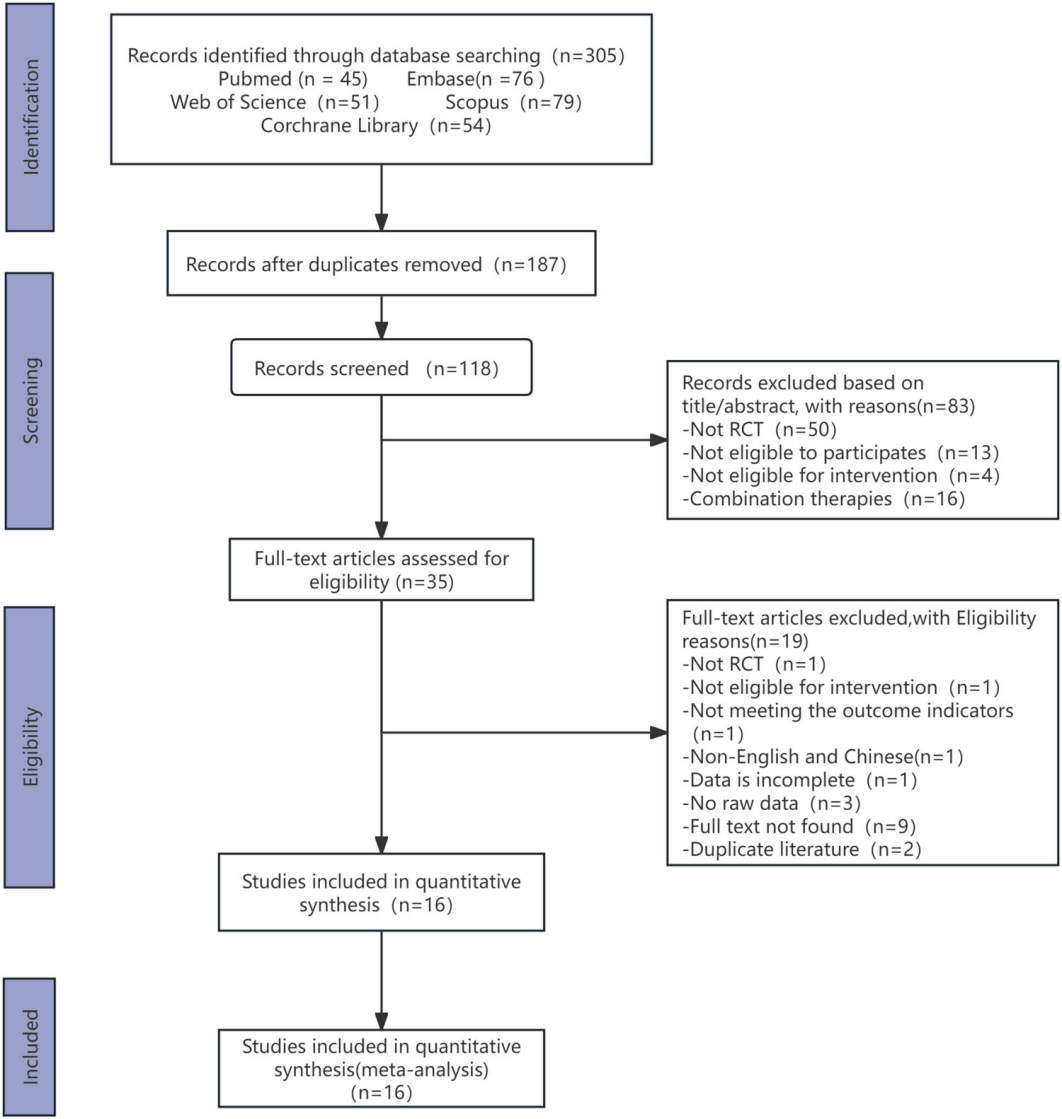
PRlSMA flow chart for study screening.

The screening flowchart is shown in Figure 1. Detailed information on excluded studies and reasons for exclusion are provided in the supplementary materials (Supplementary Material Table S4). We retrieved a total of 305 articles by searching five different databases. After importing the bibliographic records into EndNote 21 and removing duplicates, 118 articles remained. Through title and abstract screening, 83 studies failing to meet the inclusion criteria for this systematic review were excluded. Subsequently, full-text evaluation of the remaining 35 articles led to the exclusion of 19 studies for the following reasons: not a randomized controlled trial (*n* = 1); intervention not applicable (*n* = 1); outcome measures not applicable (*n* = 1); non-English or Chinese language (*n* = 1); incomplete data (*n* = 1); full text unavailable (*n* = 9); no raw data provided (*n* = 3); duplicate reference (*n* = 2). Ultimately, 16 studies were included in this meta-analysis. Among the included studies, the trial by Zahra Arjmanddoust (12)established two distinct ozone intervention groups with different concentrations (40 µg/mL and 20 µg/mL). For data analysis, this study was treated as two independent comparison groups: the higher-concentration 40 µg/mL intervention group (Group A) and the lower-concentration 20 µg/mL intervention group (Group B). Both groups were compared against the shared control group within the study and were subsequently included as independent data points in the meta-analysis.

### 3.2 Study characteristics

The basic characteristics of the included studies are presented in Table 1. This review included 16 randomized controlled trials involving 1,172 participants. These studies were conducted in Italy (*n* = 3) (13–15), Iran (*n* = 6) (12, 16–20), Brazil (*n* = 1) (21), Egypt (*n* = 2) (22, 23), Turkey (*n* = 2) (24, 25), China (*n* = 1) (26), and Spain (*n* = 1) (27). Participants were diagnosed with KOA based on ACR, KL grading, radiographic evidence, or physician confirmation.

**Table 1:** Basic characteristics of the included studies.

Author, year	Country	Patients diagnostic criteria	Mean age (year) ± SD	Sample size (IG/CG)	Intervention Group		Control group	Outcomes measure	Follow-up	Adverse effects
					Concentration/Volume	Time/Frequency				
Giombini et al. (13)	Italy	ACR	IG: 68 ± 5.36 CG: 64 ± 4.52	IG: 23 CG: 23	15 *μ*g/mL;15 mL	5 weeks/once a week	HA	VAS	at 2 months after the treatment	No adverse event
Seyed Ahmad Raeissadat et al. (16)	Iran	ACR	IG: 57.60 ± 6.1 CG: 57.91 ± 6.7	IG: 48 CG: 49	30 μg/mL;10 mL	3 weeks/once a week	HA	WOMAC Total (Pain/Function/Stiffness) VAS	at 2, 6, and 12 months after the treatment	No adverse event
Carlos Ce ar Lopes de Jesus et al. (21)	Brazil	ACR	IG: 70.5 ± 7.2 CG: 69.5 ± 7.6	IG: 61 CG: 35	20 μg/mL;10 mL	8 weeks/once a week	air	WOMAC (Pain/Function/Stiffness) VAS	2 months after the treatment	3 patients (2 in the placebo group and 1 in the ozone group) and included only puncture accidents
Zahra Arjmanddoust et al. (12)	Iran	ACR	IG1: 59.42 ± 9.93 IG2: 61.11 ± 11.05 CG: 59.68 ± 8.71	IG1: 20 IG2: 19 CG: 20	IG2:40 μg/mL;10 mL IG2:20 μg/mL;10 mL	4 weeks/once a week	O2	WOMAC Total (Pain/Function/Stiffness) VAS	at 1, 2 months after the treatment	No adverse event
Seyed Ahmad Raeissadat et al. (17)	Iran	KL scale	IG: 58.1 ± 6.4 CG: 61.1 ± 6.3	IG: 67 CG: 74	30 μg/mL;10 mL	3 weeks/once a week	HA	WOMAC Total (Pain/Function/Stiffness) VAS	6 months after the treatment	a mild flare reaction after the first injection, which was observed in five patients (2 participants in the HA group and 3 in the ozone group).
Cristiano Sconza et al. (14)	Italy	KL scale	IG: 67.5 ± 12.4 CG: 63.6 ± 13.0	IG: 56 CG: 56	10 μg/mL;10 mL	3 weeks/once a week	HA	WOMAC Total	at 1,3,6, and 12 months after the treatment	only a few mild and self-limiting adverse events occur ring within 24 h post-injection.
Cristiano Sconza et al. (15)	Italy	KL scale	IG: 68.5 ± 9.1 CG: 69.1 ± 8.7	IG: 22 CG: 22	10 μg/mL;10 mL	3 weeks/once a week	HA	WOMAC Total	at1, 3 and 6 months after the treatment	the few adverse events recorded were mild and self-limiting until 24 h after injections
Ghada Abd et al. (22)	Egypt	KL scale	IG: 44.75 ± 9.73 CG: 50.95 ± 9.47	IG:20 CG:20	10 μg/mL;10 mL	2 weeks/twice a week	dextrose	WOMAC Total	3 months after the treatment	No adverse event
Arash Babaei-Ghazani et al. (18)	Iran	ACR	IG: 59.65 ± 10.249 CG: 56.26 ± 7.887	IG: 31 CG: 31	15 μg/mL;10 mL	1 week/once a week	triamcinolone	WOMAC Total VAS	at 1, 3 months after the treatment	No adverse event
Nahla M. Gaballa et al. (23)	Egypt	ACR	IG: 56.3 ± 4.4 CG: 55.3 ± 4.2	IG:20 CG:20	25 μg/mL	4 weeks/once a week	rehabilitation	WOMAC Total VAS	at 1, 3 months after the treatment	Only minor adverse events were detected such as mild pain at injected area and skin bruises
Tahir Mutlu Duymus et al. (24)	Turkey	KL scale	IG: 59.4 ± 5.7 CG: 60.3 ± 9.1	IG: 35 CG: 34	30 μg/mL;15 mL	4 weeks/once a week	CG: HA	WOMAC Total (Pain/Function/Stiffness) VAS	at 1,3,6, and 12 months after the treatment	Apart from mild and very short-term side effects (pain, heat and redness) in a few patients, there are no side effects
Masoud Hashemi et al. (19)	Iran	KL scale	IG: 59.1 ± 12.3 CG: 57.3 ± 15.1	IG: 40 CG: 40	15 μg/mL;5–7 mL	4 weeks/Once every 10 days	hypertonic dextrose	WOMAC Total VAS	3 months after the treatment	No adverse event
Mahshid Nazarieh et al. (20)	Iran	ACR	IG: 59.58 ± 6.14 CG: 62.60 ± 5.65	IG: 19 CG: 20	20 μg/mL;10 mL	6 weeks/once a week	Placebo	VAS	at 1, 6 months after the treatment	No adverse event
Li Juan Hong et al. (26)	China	ACR	IG: 63.85 ± 1.72 CG: 64.35 ± 1.78	IG: 48 CG: 48	25 μg/mL;10–18 mL	4 weeks/once a week	Futalin Tablets	WOMAC Total (Pain/Function/Stiffness) VAS	1 month after the treatment	Except for the ozone group, transient stomach discomfort occurred in 3 to 6 cases in the other groups. It was advised to take the medication 30 min after meals, which relieved the symptoms.
Sefa Gümrük Aslan et al. (25)	Turkey	ACR	IG: 62.59 ± 9.52 CG: 62.51 ± 8.59	IG: 49 CG: 47	First: 10 μg/mL;10 mL Second: 15 μg/mL;10 mL Third: 20 μg/mL;10 mL	3 weeks/once a week	betamethasone	WOMAC Total VAS	at 1, 3 months after the treatment	No adverse event
Fernandez-Cuadros et al. (27)	Spain	KL scale	IG: 65.36 ± 11.02 CG: 58.03 ± 10.31	IG: 27 CG: 27	20 μg/mL;20 mL	4 weeks/once a week	PRP	WOMAC (Pain/Function/Stiffness) VAS	at 2 months after the treatment	No adverse event

SD, standard deviation; ACR, American college of rheumatology; IG, intervention group; CG, control group; HA, hyaluronic acid; VAS, visual analogue scale; PRP, platelet-rich plasma; WOMAC, Western Ontario and McMaster Universities Osteoarthritis index; KL scale, Kellgren–Lawrence scale.

For interventions, the intervention group received intra-articular ozone therapy without restrictions on concentration, dosage, or frequency. In the control group, 6 studies used hyaluronic acid (13–17, 24), 3 studies used placebo (12, 20, 21), 2 studies used glucose (19, 22), 2 studies used glucocorticoids (18, 25), 1 study used platelet-rich plasma(PRP) (27), 1 study used Voltaren Tablet (26), and 1 study used rehabilitation therapy (23).

Within the framework of comparing efficacy stratified by follow-up time, the distribution of studies reporting each outcome measure is as follows, For WOMAC: At 1-month follow-up, 8 studies (12, 14, 15, 18, 23–26) reported WOMAC Total; At 2-month follow-up, 3 studies (12, 16, 27) reported WOMAC pain, WOMAC stiffness, and WOMAC physical function; At 3-month follow-up, 8 studies (14, 15, 18, 19, 22–25) reported WOMAC Total; at 6-month follow-up, 5 studies (14–17, 24) reported WOMAC Total; at 12-month follow-up, 3 studies (14, 16, 24) reported WOMAC Total. Regarding VAS: At 1-month follow-up, 7 studies (12, 18, 20, 23–26) reported VAS pain; at 2-month follow-up, 5 studies (12, 13, 16, 21, 27) reported VAS pain; at 3-month follow-up, 5 studies (18, 19, 23–25) reported VAS pain; at 6-month follow-up, 4 studies (16, 17, 20, 24) reported VAS pain; at 12-month follow-up, 2 studies (16, 24) reported VAS pain.

Regarding safety, 7 studies reported minor adverse events. Among these, adverse reactions in 6 studies (14, 15, 17, 21, 23, 24) were directly related to the injection procedure itself, primarily manifesting as transient pain or localized warmth at the injection site. All symptoms resolved spontaneously within a short period and did not affect the treatment process. One additional study (26) reported a single case of gastric discomfort associated with oral medication. Symptoms resolved after adjusting the medication to be taken after meals. This event had no direct causal relationship with the ozone therapy. Overall, existing data indicate that intra-articular ozone therapy for knee osteoarthritis demonstrates good safety, with serious adverse events being rare.

### 3.3 Assessment of risk of bias

**Figure 2:**
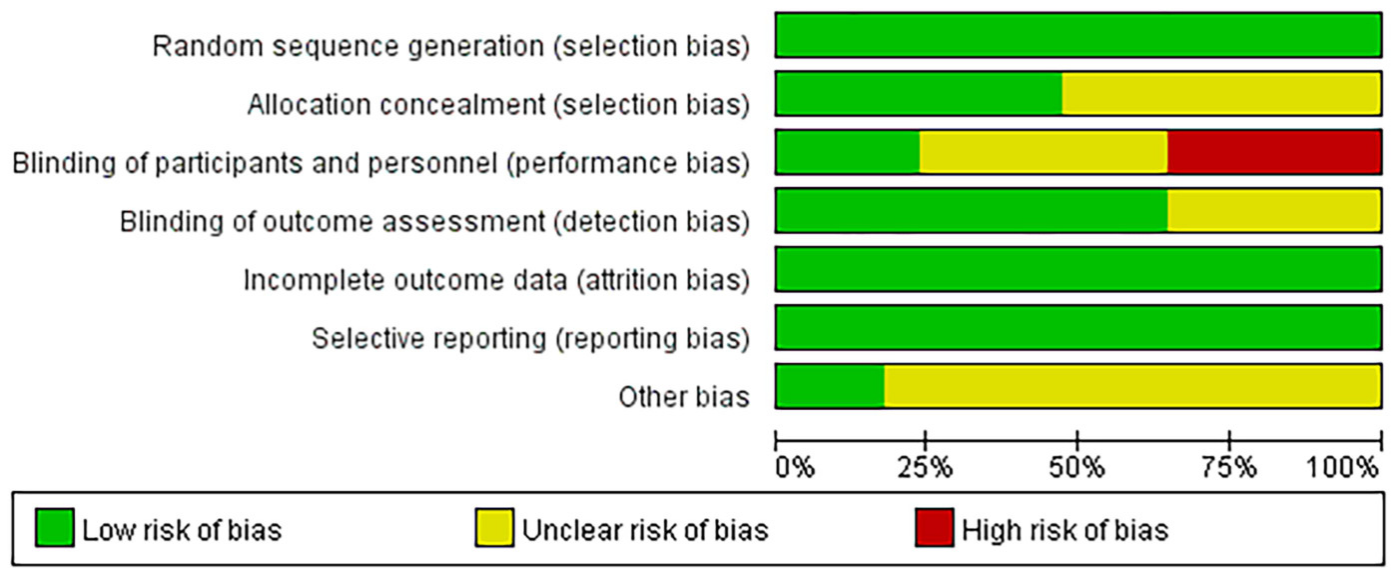
Risk of bias graph.

**Figure 3:**
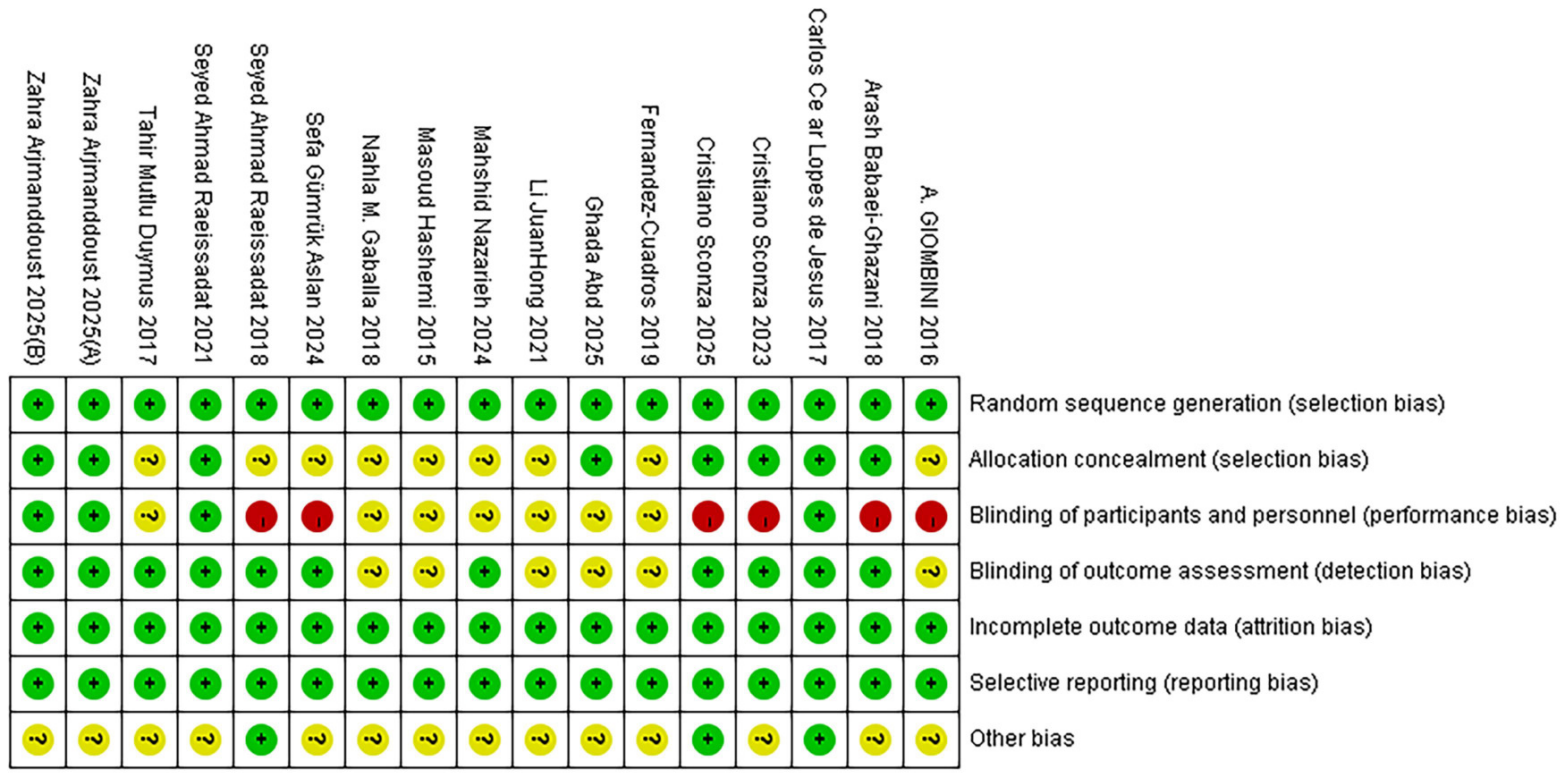
Risk of summary.

The risk of bias assessments for all included studies are summarized in Figures 2, 3, with detailed reasons for bias presented in the supplementary materials (Supplementary Material Table S5). Among the 16 included studies, all described the random sequence generation process, which was considered low risk. Within these studies, 7 reported allocation concealment methods (12, 14–16, 18, 21, 22), and 3 reported blinding of participants and researchers (12, 16, 21). Regarding outcome data completeness and blinding assessment, 16 and 10 studies (12, 14–18, 20, 21, 24, 25), respectively, demonstrated low risk. Sixteen studies reported the number of participants lost to follow-up and the reasons for loss. Finally, 13 studies showed unclear risk of other biases (12, 13, 15, 16, 18–20, 22–27), while the remaining 3 studies did not demonstrate apparent other biases (14, 17, 21).

### 3.4 Outcome of intervention

**Figure 4:**
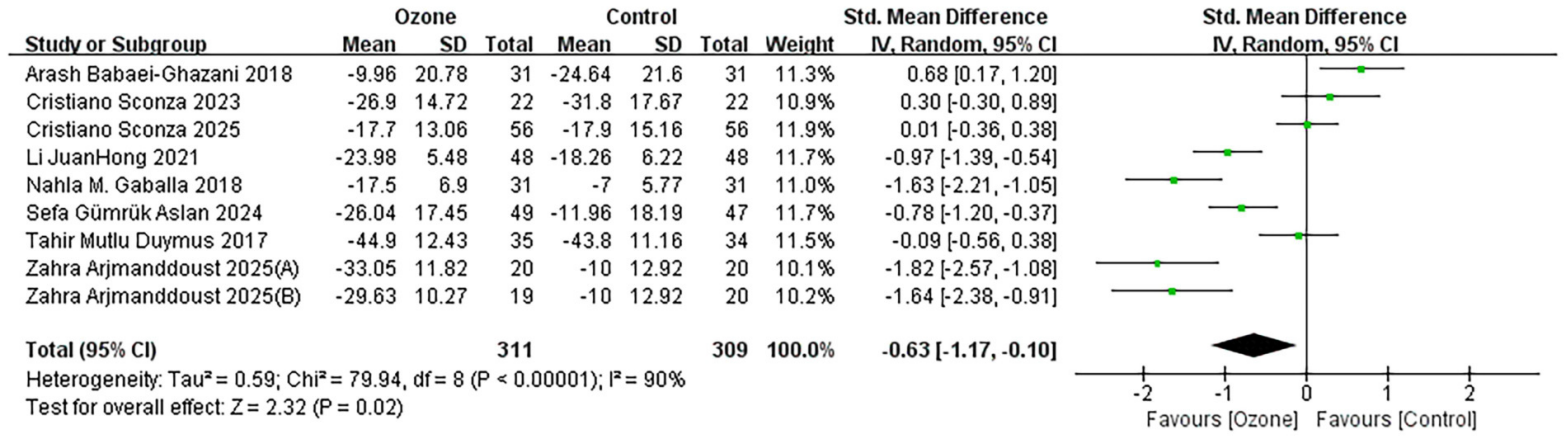
Forest plot of the impact of ozone therapy on WOMAC total T1 in patients with KOA.

**Figure 5:**
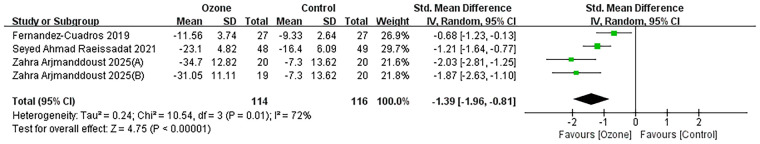
Forest plot of the impact of ozone therapy on WOMAC pain T2 in patients with KOA.

**Figure 6:**
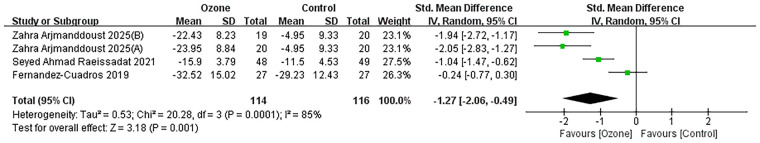
Forest plot of the impact of ozone therapy on WOMAC function T2 in patients with KOA.

**Figure 7:**
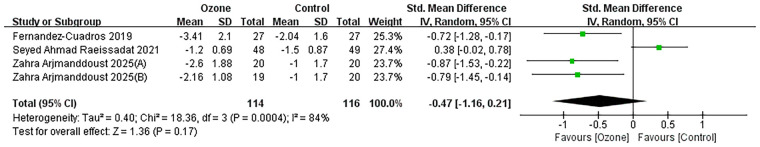
Forest plot of the impact of ozone therapy on WOMAC stiffness T2 in patients with KOA.

**Figure 8:**
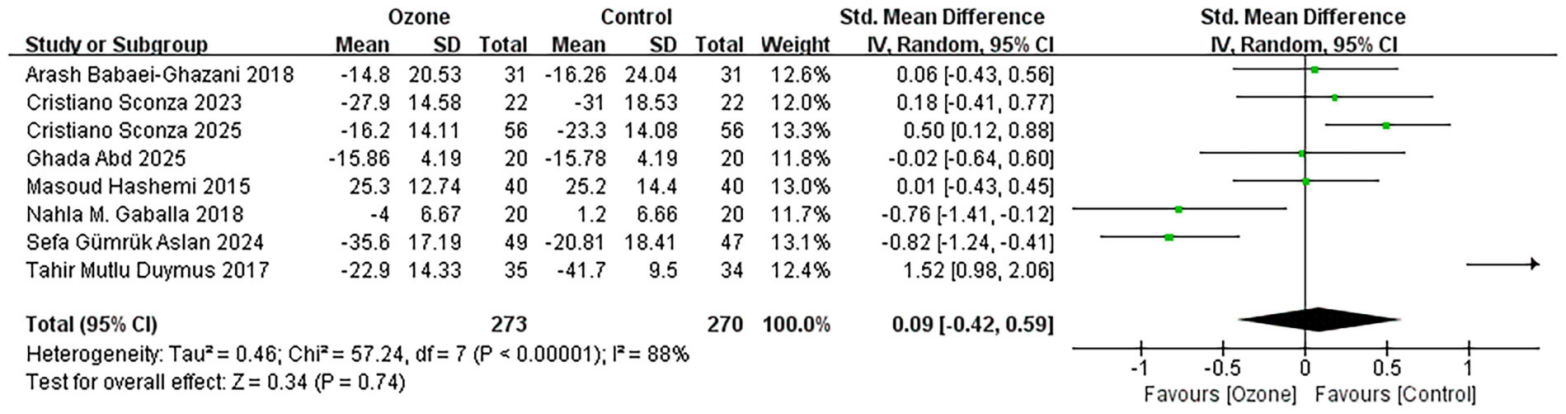
Forest plot of the impact of ozone therapy on WOMAC total T3 in patients with KOA.

**Figure 9:**
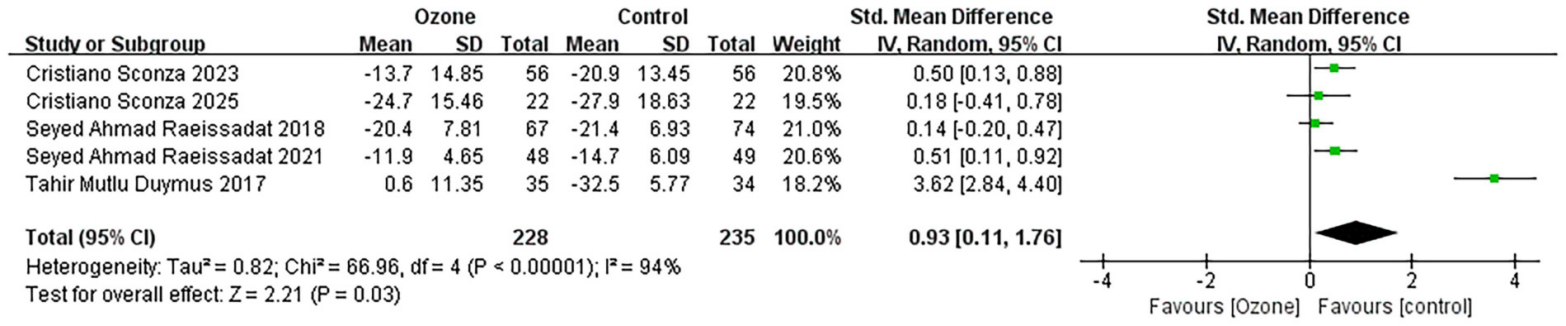
Forest plot of the impact of ozone therapy on WOMAC total T6 in patients with KOA.

**Figure 10:**
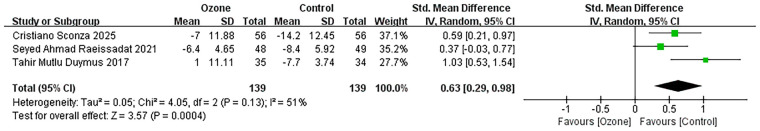
Forest plot of the impact of ozone therapy on WOMAC total T12 in patients with KOA.

**Figure 11:**
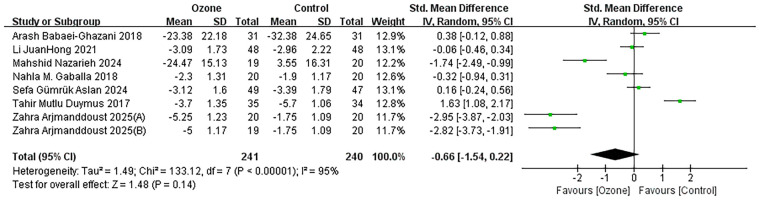
Forest plot of the impact of ozone therapy on VAS T1 in patients with KOA.

**Figure 12:**
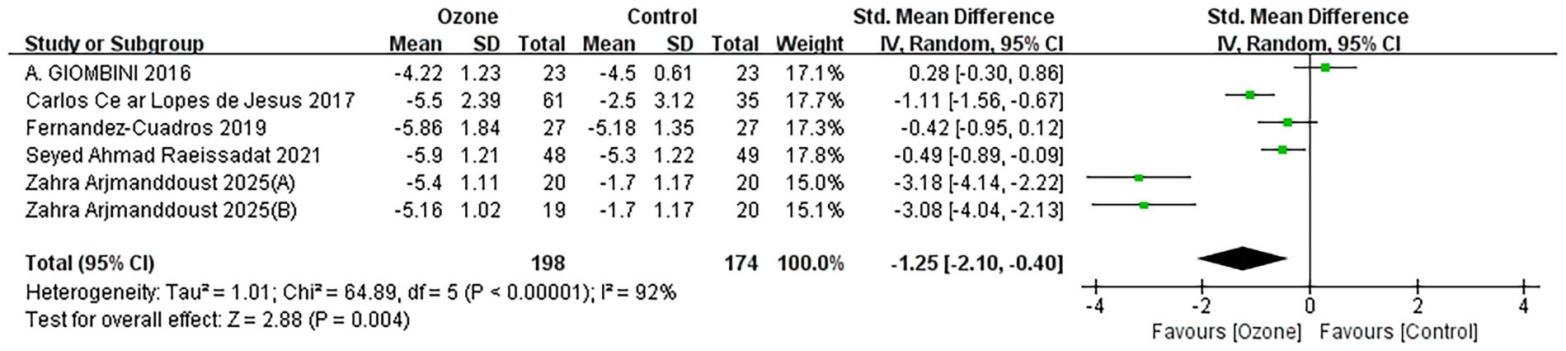
Forest plot of the impact of ozone therapy on VAS T2 in patients with KOA.

**Figure 13:**
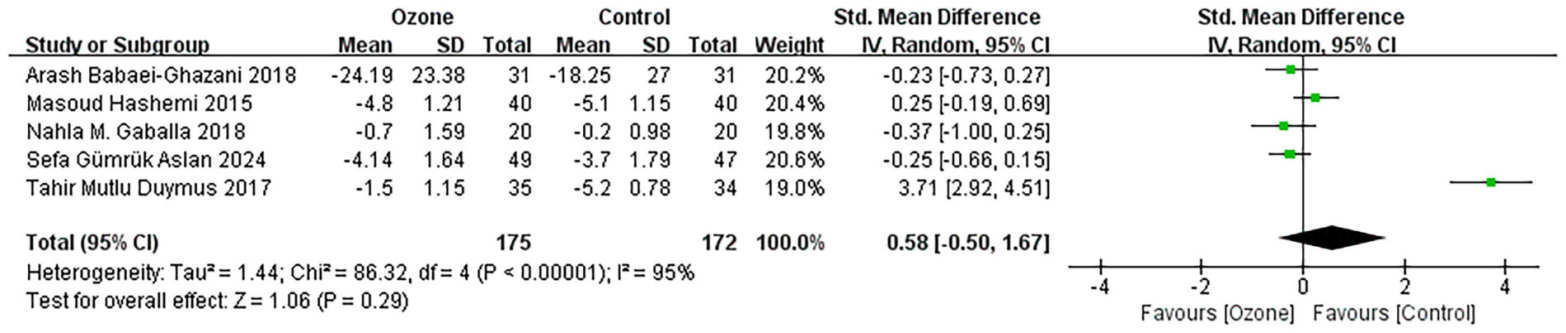
Forest plot of the impact of ozone therapy on VAS T3 in patients with KOA.

**Figure 14:**
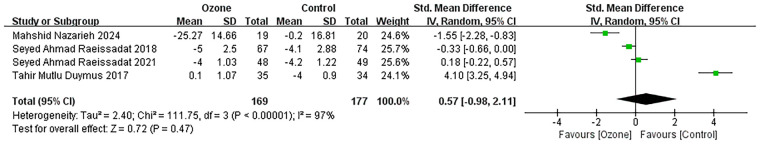
Forest plot of the impact of ozone therapy on VAS T6 in patients with KOA.

**Figure 15:**
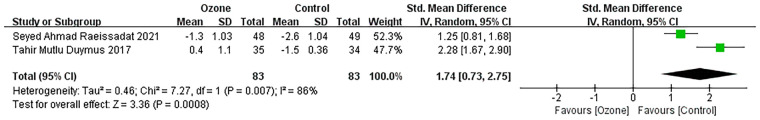
Forest plot of the impact of ozone therapy on VAS T12 in patients with KOA.

To clarify the temporal characteristics of treatment efficacy, analyses were stratified according to prespecified follow-up time points (1 month, 2 months, 3 months, 6 months, and 12 months) to compare differences in effect sizes across these time points. For each time point and corresponding outcome measure, we constructed forest plots (Figures 4–15) based on baseline and post-intervention data. Due to the use of different measurement tools, we calculated SMD and 95% CI to standardize the magnitude of outcome data.

High statistical heterogeneity (I^2^ > 75%) was observed in multiple meta-analyses, which may be attributed to differences in control interventions, ozone concentrations (10–40 µg/mL), and administration regimens (weekly injections for 3–5 weeks), as well as the diversity of the patient populations. To investigate and address this heterogeneity, we conducted *post-hoc* subgroup analyses (by type of control, ozone concentration, and regimen) and sensitivity analyses.

#### 3.4.1 WOMAC total T1

An analysis was conducted on the WOMAC Total at the 1-month follow-up for 8 studies, involving 289 participants. Results (Figure 4) showed that the ozone group demonstrated a significant reduction in the WOMAC Total—which comprehensively assesses pain, stiffness, and function—compared to the control group, reflecting the intervention's improvement in patients’ overall condition [SMD = − 0.63, 95% CI (−1.17, −0.10), *P* < 0.01]. The meta-analysis revealed high heterogeneity among studies (*I*^2^ = 90%, *P* < 0.01). Therefore, a random-effects model was employed to combine these studies.

#### 3.4.2 WOMAC pain/function/stiffness T2

Analysis of WOMAC pain, WOMAC stiffness, and WOMAC physical function at the 2-month follow-up for 3 studies included 96 participants. Results (Figures 5–7): For pain relief, the ozone group demonstrated significantly greater efficacy than the control group [SMD = − 1.39, 95% CI (−1.96, −0.81), *P* < 0.01]; For improving physical function, the ozone group showed significantly greater improvement than the control group [SMD = − 1.27, 95% CI (−2.06, −0.49), *P* < 0.01]; Regarding stiffness improvement, the meta-analysis showed a trend toward reduced WOMAC stiffness scores with ozone therapy, but its 95% confidence interval spanned the non-difference line [SMD = − 0.47, 95% CI (−1.16, 0.21), *P* < 0.01], indicating high statistical uncertainty regarding this effect. Therefore, it remains inconclusive whether ozone therapy significantly improves stiffness. The meta-analysis revealed substantial heterogeneity among studies (*I*^2^ = 72%, 85%, 84%, *P* < 0.01). Consequently, a random-effects model was employed for the meta-analysis.

#### 3.4.3 WOMAC total T3

An analysis was conducted on the WOMAC Total at the 3-month follow-up of 8 studies, involving 270 participants. Results (Figure 8) showed that the pooled effect size for WOMAC Total approached non-significance, with a wide 95% confidence interval spanning zero [SMD = 0.09, 95% CI (−0.42, 0.59), *P* < 0.01]. This indicates that ozone therapy provided no significant improvement in overall patient symptoms and function at this time point. Overall results demonstrated high heterogeneity among studies (*I*^2^ = 88%, *P* < 0.01). Therefore, a random-effects model was employed for the meta-analysis.

#### 3.4.4 WOMAC total T6

An analysis of the WOMAC Total at the 6-month follow-up across 5 studies involving 235 participants revealed (Figure 9) a pooled effect size of SMD = 0.93 for WOMAC Total. The confidence interval for this effect size was entirely to the right of the null line and did not include zero [SMD = 0.93, 95% CI (0.11, 1.76)]. This indicates that patients in the ozone therapy group had significantly higher overall symptom and functional scores compared to the control group, suggesting ozone therapy no longer provided improvement at this time point. Conversely, the results tended to support the control group achieving relatively superior overall efficacy. Overall results showed high heterogeneity among studies (*I*^2^ = 94%, *P* < 0.01). Thus, we employed a random-effects model for the meta-analysis.

#### 3.4.5 WOMAC total T12

Analysis of WOMAC Total at the 12-month follow-up across 3 studies included 139 participants. Results (Figure 10) showed a pooled effect size for WOMAC Total of SMD = 0.63, with its confidence interval entirely to the right of the null line and excluding zero [SMD = 0.63, 95% CI (0.29, 0.98)]. This finding indicates that at the long-term follow-up endpoint, the ozone therapy group still exhibited significantly higher overall symptom and functional scores than the control group, further confirming the lack of sustained efficacy of ozone therapy. Conversely, the direction of this effect suggests that the control group may have superior long-term clinical outcomes. Overall results showed high heterogeneity among studies (*I*^2^ = 51%, *P* < 0.01). Hence, a random-effects model was employed for meta-analysis.

#### 3.4.6 VAS T1

Analysis of VAS pain at the 1-month follow-up from 7 studies involving 220 participants The results (Figure 11) indicate that while ozone therapy showed a trend toward pain reduction, the effect was not statistically certain. It cannot be concluded that it significantly improves pain at 1 month [SMD = − 0.66, 95% CI (−1.54, 0.22)]. Overall results demonstrated high heterogeneity among studies (*I*^2^ = 95%, *P* < 0.01). As such, we employed a random-effects model for the meta-analysis of these studies.

#### 3.4.7 VAS T2

Analysis of VAS pain at the 2-month follow-up from 5 studies involving 164 participants revealed (Figure 12) that the ozone group significantly outperformed the control group at this time point, demonstrating a marked reduction in patient pain [SMD = − 1.25, 95% CI (−2.10, −0.40)]. Overall results demonstrated high heterogeneity among studies (*I*^2^ = 92%, *P* < 0.01). As a result, we employed a random-effects model for the meta-analysis of these studies.

#### 3.4.8 VAS T3

An analysis of VAS pain at the 3-month follow-up across 5 studies involving 172 participants revealed (Figure 13) a pooled effect size of SMD = 0.58, with a wide confidence interval spanning the null line [SMD = 0.58, 95% CI (−0.50, 1.67)]. This finding suggests that the significant analgesic effect observed at 2 months with ozone therapy had disappeared by this time point. The direction of the point estimate even indicated a trend toward higher pain scores compared to the control group; however, since the confidence interval included zero, this effect was statistically uncertain, and no definitive conclusion could be drawn. Overall results demonstrated high heterogeneity among studies (*I*^2^ = 95%, *P* < 0.01). for this reason, we employed a random-effects model for the meta-analysis.

#### 3.4.9 VAS T6

Analysis of VAS pain at the 6-month follow-up across 4 studies involving 177 participants revealed (Figure 14) a pooled effect size of SMD = 0.57. The confidence interval spanned a wide range and crossed the null line, indicating high imprecision in this effect estimate [SMD = 0.57, 95% CI (−0.98, 2.11)]. Although the direction of the point estimate suggests that the ozone therapy group may have higher pain scores than the control group, the substantial uncertainty in the statistical evidence precludes drawing a conclusion about a clear clinical effect at this time point. Overall results showed high heterogeneity among studies (*I*^2^ = 97%, *P* < 0.01). Accordingly, we employed a random-effects model for the meta-analysis of these studies.

#### 3.4.10 VAS T12

Analysis of VAS pain at the 12-month follow-up for 2 studies involving 83 participants revealed (Figure 15) a pooled effect size of SMD = 1.74. Its confidence interval fell entirely to the right of the null line and excluded zero [SMD = 1.74, 95% CI (0.73, 2.75)]. This finding indicates that patients in the ozone therapy group had significantly higher pain scores compared to the control group, suggesting that ozone failed to sustain analgesic effects at long-term follow-up endpoints. This may indicate that the therapeutic effects of ozone therapy do not last over the long term. Overall results revealed high heterogeneity among studies (*I*^2^ = 86%, *P* < 0.01). Therefore, we employed a random-effects model for the meta-analysis of these studies.

### 3.5 GRADE evidence quality assessment

The GRADEpro GDT was used to assess the quality of evidence for outcome measures at different follow-up times (based on risk of bias, inconsistency, indirectness, imprecision, and publication bias), preliminary results (Table 2) indicate that among 12 analyses, 4 were rated as moderate quality, 5 as low quality, and 3 as very low quality. Notably, evidence suggesting the efficacy of ozone therapy at short-term follow-up (1 to 2 months) was of relatively higher quality. For example, The body of evidence for the WOMAC pain score (2-month follow-up), WOMAC function score (2-month follow-up), WOMAC total score (1-month follow-up), and VAS score (2-month follow-up) was rated as moderate quality. These findings indicate that existing evidence demonstrates high credibility regarding ozone therapy's ability to improve short-term pain, function, and overall symptoms in patients with knee osteoarthritis.

**Table 2:** GRADE results for outcome measures at each follow-up time point.

WOMAC for [KOA]						
Patient or population: patients with [KOA] Settings: Intervention: WOMAC						
Outcomes	Illustrative comparative risks* (95% CI)		Relative effect (95% CI)	No of Participants (studies)	Quality of the evidence (GRADE)	Comments
	Assumed risk	Corresponding risk				
	Control	WOMAC				
**WOMAC Total T1** Follow-up: mean 1 months		The mean womac total t1 in the intervention groups was **0.63 standard deviations lower** (1.17 to 0.1 lower)		620 (9 studies)	⊕⊕⊕⊝ **moderate**^a^	SMD −0.63 (−1.17 to −0.1)
**WOMAC Pain T2** Follow-up: mean 2 months		The mean womac pain t2 in the intervention groups was **1.39 standard deviations lower** (1.96 to 0.81 lower)		230 (4 studies)	⊕⊕⊕⊝ **moderate**^a^	SMD −1.95 (−2.49 to −1.4)
**WOMAC Function T2** Follow-up: mean 2 months		The mean womac function t2 in the intervention groups was **1.27 standard deviations lower** (2.06 to 0.49 lower)		230 (4 studies)	⊕⊕⊕⊝ **moderate**^a^	SMD −2 (−2.55 to −1.45)
**WOMAC Stiffness T2** Follow-up: mean 2 months		The mean womac stiffness t2 in the intervention groups was **0.47 standard deviations lower** (1.16 lower to 0.21 higher)		230 (4 studies)	⊕⊕⊝⊝ **low**^a^^,^^b^	SMD −0.47 (−1.16 to 0.21)
**WOMAC Total T3** ollow-up: mean 3 months		The mean womac total t3 in the intervention groups was **0.09 standard deviations higher** (0.42 lower to 0.59 higher)		543 (8 studies)	⊕⊕⊝⊝ **low**^a^^,^^b^	SMD 0.2 (−0.02 to 0.41)
**WOMAC Total T6** Follow-up: mean 6 months		The mean womac total t6 in the intervention groups was **0.93 standard deviations higher** (0.11 to 1.76 higher)		463 (5 studies)	⊕⊕⊝⊝ **low**^a^^,^^b^	SMD 0.34 (0.14 to 0.54)
**WOMAC Total T12** Follow-up: mean 12 months		The mean womac total t12 in the intervention groups was **0.63 standard deviations higher** (0.29 to 0.98 higher)		278 (3 studies)	⊕⊕⊝⊝ **low**^a^^,^^b^	SMD 0.49 (0.21 to 0.76)

VAS for [KOA]						
Patient or population: patients with [KOA] Settings: Intervention: VAS						
Outcomes	Illustrative comparative risks* (95% CI)		Relative effect (95% CI)	No of participants (studies)	Quality of the evidence (GRADE)	Comments
	Assumed risk	Corresponding risk				
	Control	VAS				
**VAS T1** Follow-up: mean 1 months		The mean vas t1 in the intervention groups was **0.66 standard deviations lower** (1.54 lower to 0.22 higher)		481 (8 studies)	⊕⊝⊝⊝ **very low**^a^^,^^b^^,^^c^	SMD −0.66 (−1.54 to 0.22)
**VAS T2** Follow-up: mean 2 months		The mean vas t2 in the intervention groups was **1.25 standard deviations lower** (2.1 to 0.4 lower)		372 (6 studies)	⊕⊕⊕⊝ **moderate**^b^	SMD −1.25 (−2.1 to −0.4)
**VAS T3** Follow-up: mean 3 months		The mean vas t3 in the intervention groups was **0.58 standard deviations higher** (0.5 lower to 1.67 higher)		347 (5 studies)	⊕⊝⊝⊝ **very low**^a^^,^^b^^,^^c^	SMD 0.58 (−0.5 to 1.67)
**VAS T6** Follow-up: mean 6 months		The mean vas t6 in the intervention groups was **0.57 standard deviations higher** (0.98 lower to 2.11 higher)		346 (4 studies)	⊕⊝⊝⊝ **very low**^a^^,^^b^^,^^c^	SMD 0.57 (−0.98 to 2.11)
**VAS T12** Follow-up: mean 12 months		The mean vas t12 in the intervention groups was **1.74 standard deviations higher** (0.73 to 2.75 higher)		166 (2 studies)	⊕⊕⊝⊝ **low**^b^^,^^c^	SMD 1.74 (0.73 to 2.75)

GRADE Working Group grades of evidence.

High quality: Further research is very unlikely to change our confidence in the estimate of effect.

Moderate quality: Further research is likely to have an important impact on our confidence in the estimate of effect and may change the estimate.

Low quality: Further research is very likely to have an important impact on our confidence in the estimate of effect and is likely to change the estimate.

Very low quality: We are very uncertain about the estimate.

* The basis for the assumed risk (e.g., the median control group risk across studies) is provided in footnotes. The **corresponding risk** (and its 95% confidence interval) is based on the assumed risk in the comparison group and the relative effect of the intervention (and its 95% CI). CI, confidence interval.

a The studies included in the analysis exhibited bias in terms of randomization, allocation concealment, and blinding.

b The sample size included in the study is too small, or the confidence interval is too wide.

GRADE Working Group grades of evidence.

High quality: Further research is very unlikely to change our confidence in the estimate of effect.

Moderate quality: Further research is likely to have an important impact on our confidence in the estimate of effect and may change the estimate.

Low quality: Further research is very likely to have an important impact on our confidence in the estimate of effect and is likely to change the estimate.

Very low quality: We are very uncertain about the estimate.

* The basis for the assumed risk (e.g., the median control group risk across studies) is provided in footnotes. The **corresponding risk** (and its 95% confidence interval) is based on the assumed risk in the comparison group and the relative effect of the intervention (and its 95% CI). CI, confidence interval.

a The studies included in the analysis exhibited bias in terms of randomization, allocation concealment, and blinding.

b Results with significant heterogeneity and currently unexplained

c The sample size included in the study is too small, or the confidence interval is too wide.

### 3.6 Sensitivity analysis

We conducted a sensitivity analysis of WOMAC and VAS at different follow-up time points to assess the robustness of the overall results. For all time points and outcome measures, no single study had a disproportionate influence on the overall effect estimate, as the pooled standardized mean difference (SMD) remained within the 95% confidence interval (CI) of the original analysis (Supplementary Figure S2 in the Supplementary Materials), indicating that the findings remain robust despite high statistical heterogeneity.

## 4 Discussion

### 4.1 Overall findings

The mechanism of ozone therapy for knee osteoarthritis primarily relies on its induction of controlled acute oxidative stress. Upon entering the joint, ozone decomposes to produce reactive oxygen species (ROS) and lipid oxidation products (LOPS). These messenger substances activate endogenous antioxidant systems (such as superoxide dismutase and glutathione peroxidase), thereby alleviating chronic oxidative damage within the joint (5, 28). Simultaneously, ozone modulates inflammatory responses by suppressing the release of pro-inflammatory cytokines (e.g., IL-1β, TNF-α) and potentially promoting the expression of anti-inflammatory cytokines (e.g., IL-4, IL-10, TGF-β), thereby reducing synovial inflammation (29). These combined biological effects may explain its clinical efficacy in providing short-term pain relief and improving joint function.

This study systematically evaluated the time-dependent efficacy of intra-articular ozone therapy for knee osteoarthritis through systematic reviews and meta-analyses at different follow-up time points. Key findings can be summarized as follows: Ozone therapy can significantly improve pain, function, and overall symptoms in the short term (1–2 months), but its benefits may not be sustained over the long term. This time-dependent effect aligns with existing clinical observations and conclusions from some studies, indicating that ozone therapy exhibits rapid onset but limited duration of action (13, 14, 16).

Regarding the mechanism behind the time-dependent decline in therapeutic efficacy, we hypothesize that it may be related to the unique characteristics of ozone therapy. Ozone treatment primarily induces controlled oxidative stress, thereby activating the endogenous antioxidant system and modulating inflammatory responses. This biological effect is likely transient and reversible. As the intra-articular microenvironment gradually returns to baseline oxidative stress levels, coupled with the progressive pathological process inherent to knee osteoarthritis itself, the therapeutic effects become difficult to sustain.

This study observed high heterogeneity (*I*^2^ > 50%) across all analyses. Although results were pooled using a random-effects model and sensitivity analyses confirmed their robustness, thoroughly examining the sources of heterogeneity is crucial for accurate interpretation. Through a series of *post-hoc* subgroup analyses, we successfully identified several key factors influencing WOMAC heterogeneity (Supplementary Material Figure S1): First, differences in control group interventions were the primary source of short-term efficacy heterogeneity. For example, when studies where the control group received hyaluronic acid injections were analyzed separately at the 1-month follow-up, heterogeneity decreased to negligible levels. Similarly, at the 2-month follow-up, studies using medical oxygen as the control also demonstrated high internal consistency. This indicates that the efficacy differences inherent in the various control measures significantly influenced the effect size estimates when compared to ozone therapy. Second, variations in ozone intervention parameters were the core cause of medium-to-long-term efficacy heterogeneity. At the 3-month follow-up, stratification by ozone concentration ≤ 20 µg/mL resolved heterogeneity. At the 6-month and 12-month follow-ups, differences in injection regimens (e.g., “once weekly for 3 consecutive weeks” vs. other regimens) were confirmed as key drivers of inconsistent outcomes. These findings strongly suggest that ozone concentration and treatment frequency are key variables determining the magnitude of short-term effects and heterogeneity across studies. However, regardless of concentration or treatment duration, the therapeutic effects of ozone disappeared after 3 months, suggesting that long-term maintenance of efficacy cannot be achieved within the current range of parameters. Although we conducted *post-hoc* subgroup analyses to explore the sources of heterogeneity (Supplementary Figure S1 in the Supplementary Materials), we acknowledge that, due to the limited number of available studies at each follow-up time point (fewer than 10), we did not perform a formal meta-regression analysis, as this would have resulted in insufficient statistical power and could have been misleading. However, in the VAS analysis, despite exploratory analyses accounting for known factors (control type, ozone concentration, injection regimen) and potential additional clinical variables, no single dominant factor explaining the high heterogeneity was identified. This suggests that VAS heterogeneity may stem from a more complex combination of confounding factors.

Given the heterogeneity of the control groups, subgroup analyses were performed for all key time points of WOMAC and VAS by control type (placebo/oxygen vs. hyaluronic acid). These results are presented in the new Supplementary Material Figure S3. Key findings: Ozone demonstrated superior absolute efficacy compared to placebo in the short term, but showed no advantage over HA in the medium to long term and was even inferior; the long-term “inferiority of ozone” was primarily due to the active controls (such as HA) having more sustained efficacy, rather than any harmful effects of ozone itself.To investigate whether the positive combined effect observed at 6 and 12 months might be due to the inclusion of active comparators with known long-term efficacy (hyaluronic acid, platelet-rich plasma, corticosteroids), we conducted a sensitivity analysis in which we excluded all such studies. At 6 months, no studies remained for the WOMAC total score after exclusion, as all five trials used hyaluronic acid as a control. For the VAS at 6 months, one placebo-controlled trial (Nazarieh 2024) was available; whose effect size [standardized mean difference [SMD] = − 1.79, 95% confidence interval [CI] [−2.52, −1.06]] strongly favored ozone therapy, contrasting with the positive pooled estimate from the full analysis [SMD = 0.57, 95% CI (−0.98, 2.11)]. At 12 months, no placebo-controlled studies were available for either outcome measure. These findings suggest that the positive pooled estimate at long-term follow-up is primarily attributable to the superior efficacy of active comparators rather than to harmful effects of ozone, consistent with the results mentioned in the subgroup analysis above. However, the number of long-term placebo-controlled trials is extremely limited, so definitive conclusions cannot be drawn.

Despite the increasing application of ozone therapy in knee osteoarthritis in recent years, its overall evidence base remains weak. Sconza (5) noted that relevant randomized controlled trials generally suffer from low methodological quality, high risk of bias, and inconsistencies in treatment protocols (such as concentration, dosage, and injection frequency). This aligns with our analysis of sources of heterogeneity and underscores the urgent need for standardized treatment protocols in the future.

### 4.2 Limitations and future research directions

This study has several limitations. First, constraints exist in both the quantity and quality of the evidence base. Although we included 16 randomized controlled trials, high-quality research on ozone therapy remains limited compared to other established treatments for knee osteoarthritis. In particular, the scarcity of studies with long-term follow-up (e.g., 12 months) may compromise the stability and certainty of conclusions regarding long-term efficacy. Additionally, the overall methodological quality of the included studies was moderate, with deficiencies in allocation concealment and blinding that may have introduced bias. Second, significant clinical and methodological heterogeneity existed. Substantial variations in ozone concentration, injection volume, treatment frequency, and control group interventions across studies were the primary drivers of the high heterogeneity observed. Although we employed a random-effects model and conducted subgroup analyses to explore sources of heterogeneity—successfully explaining most of the heterogeneity in WOMAC—the heterogeneity in VAS could not be fully resolved. This suggests the presence of more complex confounding factors that may have influenced the precise interpretation of the pooled results for this outcome measure. Third, since fewer than 10 studies were included at all follow-up time points in this meta-analysis, funnel plots and Egger's tests are methodologically recommended to be avoided due to low power and extreme instability of results. Therefore, quantitative or graphical assessments of publication bias were not performed to prevent misleading conclusions. Nevertheless, the lack of any assessment of publication bias remains one of the limitations of this study. The existing evidence is subject to the following potential risks of bias: First, the small-study effect—the 16 randomized controlled trials included in this review had a total sample size of 1,172 participants, with an average of only about 73 participants per trial. Small studies tend to show larger effect sizes (especially in the absence of strict blinding) and are more susceptible to random errors. Second, the possibility of unpublished negative trials—due to publication bias, studies with negative or ineffective results may not have been published, leading us to overestimate the efficacy of ozone. This is particularly evident in short-term (1–2 months) placebo-controlled studies, as the results of these studies were all significantly in favor of ozone, and any unpublished negative studies would alter this conclusion. Therefore, when interpreting the pooled effect sizes from this study, readers should consider the potential overestimation of effects due to publication bias.Fourth, the selection of outcome measures and follow-up points may not be comprehensive. This study primarily focused on patient-reported pain and functional outcomes (WOMAC,VAS) without systematically evaluating imaging progression, inflammatory biomarker changes, or long-term safety data. Additionally, analyses were based on discrete time points (1, 2, 3, 6, 12 months), failing to depict the dynamic curve of continuous efficacy decline. Fifth, the long-term evidence (6 and 12 months) comes almost exclusively from studies using active comparators known to have sustained efficacy (hyaluronic acid, platelet-rich plasma). To date, there are no long-term trials with sham surgery controls, and none of the included studies evaluated the frequency of repeated ozone therapy. Therefore, the finding that the control group performed better than the ozone treatment group at 12 months should not be interpreted as evidence of harm, but rather reflects the relative disadvantage of a single course of ozone therapy compared to long-acting active treatments.

Given these limitations, this paper proposes several directions for future research. First, there is an urgent need for high-quality, double-blind, placebo-controlled randomized controlled trials (RCTs) with extended follow-up periods (≥12 months) to determine the absolute efficacy and safety of intra-articular ozone therapy. Such trials should standardize ozone concentration (e.g., 20–30 μg/mL), injection dose, and frequency to reduce heterogeneity. Second, future studies should employ multi-course treatment regimens (e.g., injections every 3 months) to determine whether sustained symptom control can be achieved. Direct comparisons between single-course and multi-course regimens, as well as comparisons with other long-acting therapies (e.g., hyaluronic acid, platelet-rich plasma), are crucial for clinical decision-making. Third, researchers should prospectively register trial protocols, report results at standardized time points (1, 2, 3, 6, and 12 months), and provide individual participant data to enable meta-regression analysis and more sophisticated heterogeneity analysis. The use of the Core Outcome Set for Knee Osteoarthritis is encouraged. Fourth, given the diversity of active comparators in existing trials, network meta-analyses may be considered once sufficient data have been accumulated. This would allow for indirect comparisons of ozone with multiple active treatments while preserving randomization. Fifth, future trials should incorporate objective outcome measures (e.g., imaging studies, biomarkers) in addition to patient-reported outcomes to better capture the biological effects of ozone and assess its long-term safety, particularly regarding potential oxidative stress or effects on cartilage. Finally, to mitigate publication bias, journals should encourage the publication of negative or inconclusive results, and researchers should make every effort to disseminate all trial results, regardless of the direction or magnitude of the effect.

## 5 Conclusions

This systematic review and meta-analysis systematically evaluated the time-dependent efficacy of intra-articular ozone injection therapy for knee osteoarthritis at different follow-up time points. The results indicate that a single course of ozone therapy is effective in alleviating pain and improving physical function in the short term (1–2 months), with the most significant effects observed 2 months after injection. The quality of evidence is moderate to low, downgraded primarily due to risk of bias and inconsistency. However, the therapeutic effect diminishes after 3 months, and at 6 or 12 months, outcomes are inferior to those of the active control group. At later time points, the pooled effect sizes for multiple outcomes (e.g., WOMAC total score at 6 months, VAS at 12 months) showed that scores in the ozone group were significantly worse than those in the control group, which primarily received active controls with known long-term benefits (e.g., hyaluronic acid, platelet-rich plasma).

Based on the observed time-dependent relationship, we propose the following hypothesis: if future studies evaluate repeated injections, an interval of approximately 3 months may be a reasonable starting point for exploration. It is important to note that none of the included studies evaluated repeated ozone therapy regimens; therefore, this proposal remains speculative. Future randomized controlled trials should specifically test repeated-dose regimens (e.g., injections every 3 months) and compare them with single-course ozone therapy, sham injections, or other long-acting therapies to determine whether sustained symptom control can be achieved. Currently, clinicians should consider ozone therapy as an option for short-term symptom relief in knee osteoarthritis; decisions regarding repeated injections lack evidence and should be made cautiously on an individualized basis.

## Data Availability

The original contributions presented in the study are included in the article/Supplementary Material, further inquiries can be directed to the corresponding author.
