# Supplementary material for: The time-effect relationship of intra-articular ozone injection for knee osteoarthritis: a systematic review and meta-analysis
Source: Front Pain Res (Lausanne). 2026 Jun 17;7:1843756. doi: 10.3389/fpain.2026.1843756 (PMC13319046; doi:10.3389/fpain.2026.1843756)
Supplement: Supplementary file 1 [file Datasheet2.zip › Supplementary files/Supplementary Material.docx]

**The time-effect relationship of intra-articular ozone injection for knee osteoarthritis：A systematic review and meta-analysis**

Content

**S1 Table. PRISMA 2**

**S2 Table. Search strategy 5**

**S3 Table. Raw data and calculation of mean and SD for all RCTs 7**

**S4 Table. Excluded studies and reasons 8**

**S5 Table. Reasons for risk of bias 11**

**S1 Figure. Analysis of Heterogeneity Causes 13**

**S2 Figure. Sensitivity Analysis 15**

**S3 Figure. Subgroup analyses of WOMAC and VAS outcomes stratified by control type (placebo/oxygen vs. hyaluronic acid) at different follow-up time points 21**

**S1 Table. PRISMA**

| **Section and Topic** | **Item #** | **Checklist item** | **Location where item is reported** |
| --- | --- | --- | --- |
| **TITLE** | | |  |
| Title | 1 | Identify the report as a systematic review. | Title |
| **ABSTRACT** | | |  |
| Abstract | 2 | See the PRISMA 2020 for Abstracts checklist. | Abstract |
| **INTRODUCTION** | | |  |
| Rationale | 3 | Describe the rationale for the review in the context of existing knowledge. | Introduction |
| Objectives | 4 | Provide an explicit statement of the objective(s) or question(s) the review addresses. | Introduction |
| **METHODS** | | |  |
| Eligibility criteria | 5 | Specify the inclusion and exclusion criteria for the review and how studies were grouped for the syntheses. | Inclusion and Exclusion Criteria,Statistical Analysis |
| Information sources | 6 | Specify all databases, registers, websites, organisations, reference lists and other sources searched or consulted to identify studies. Specify the date when each source was last searched or consulted. | Methods,Search Strategy |
| Search strategy | 7 | Present the full search strategies for all databases, registers and websites, including any filters and limits used. | Search Strategy |
| Selection process | 8 | Specify the methods used to decide whether a study met the inclusion criteria of the review, including how many reviewers screened each record and each report retrieved, whether they worked independently, and if applicable, details of automation tools used in the process. | Study Selection |
| Data collection process | 9 | Specify the methods used to collect data from reports, including how many reviewers collected data from each report, whether they worked independently, any processes for obtaining or confirming data from study investigators, and if applicable, details of automation tools used in the process. | Data Extraction |
| Data items | 10a | List and define all outcomes for which data were sought. Specify whether all results that were compatible with each outcome domain in each study were sought (e.g. for all measures, time points, analyses), and if not, the methods used to decide which results to collect. | Data Extraction |
|  | 10b | List and define all other variables for which data were sought (e.g. participant and intervention characteristics, funding sources). Describe any assumptions made about any missing or unclear information. | Data Extraction |
| Study risk of bias assessment | 11 | Specify the methods used to assess risk of bias in the included studies, including details of the tool(s) used, how many reviewers assessed each study and whether they worked independently, and if applicable, details of automation tools used in the process. | Risk of bias assessment and GRADE |
| Effect measures | 12 | Specify for each outcome the effect measure(s) (e.g. risk ratio, mean difference) used in the synthesis or presentation of results. | Statistical Analysis |
| Synthesis methods | 13a | Describe the processes used to decide which studies were eligible for each synthesis (e.g. tabulating the study intervention characteristics and comparing against the planned groups for each synthesis (item #5)). | Statistical Analysis |
|  | 13b | Describe any methods required to prepare the data for presentation or synthesis, such as handling of missing summary statistics, or data conversions. | Statistical Analysis |
|  | 13c | Describe any methods used to tabulate or visually display results of individual studies and syntheses. | Statistical Analysis |
|  | 13d | Describe any methods used to synthesize results and provide a rationale for the choice(s). If meta-analysis was performed, describe the model(s), method(s) to identify the presence and extent of statistical heterogeneity, and software package(s) used. | Statistical Analysis |
|  | 13e | Describe any methods used to explore possible causes of heterogeneity among study results (e.g. subgroup analysis, meta-regression). | Statistical Analysis |
|  | 13f | Describe any sensitivity analyses conducted to assess robustness of the synthesized results. | Statistical Analysis |
| Reporting bias assessment | 14 | Describe any methods used to assess risk of bias due to missing results in a synthesis (arising from reporting biases). | Assessment of Reporting Quality |
| Certainty assessment | 15 | Describe any methods used to assess certainty (or confidence) in the body of evidence for an outcome. | Statistical Analysis |
| **RESULTS** | | |  |
| Study selection | 16a | Describe the results of the search and selection process, from the number of records identified in the search to the number of studies included in the review, ideally using a flow diagram. | Search Results |
|  | 16b | Cite studies that might appear to meet the inclusion criteria, but which were excluded, and explain why they were excluded. | Search Results |
| Study characteristics | 17 | Cite each included study and present its characteristics. | Study Characteristics |
| Risk of bias in studies | 18 | Present assessments of risk of bias for each included study. | Assessment of Risk of Bias |
| Results of individual studies | 19 | For all outcomes, present, for each study: (a) summary statistics for each group (where appropriate) and (b) an effect estimate and its precision (e.g. confidence/credible interval), ideally using structured tables or plots. | Outcome of Intervention |
| Results of syntheses | 20a | For each synthesis, briefly summarise the characteristics and risk of bias among contributing studies. | Study Characteristics，Assessment of Risk of Bias |
|  | 20b | Present results of all statistical syntheses conducted. If meta-analysis was done, present for each the summary estimate and its precision (e.g. confidence/credible interval) and measures of statistical heterogeneity. If comparing groups, describe the direction of the effect. | Outcome of Intervention |
|  | 20c | Present results of all investigations of possible causes of heterogeneity among study results. | Overall Findings |
|  | 20d | Present results of all sensitivity analyses conducted to assess the robustness of the synthesized results. | Sensitivity Analysis |
| Reporting biases | 21 | Present assessments of risk of bias due to missing results (arising from reporting biases) for each synthesis assessed. | Limitations and future research  directions |
| Certainty of evidence | 22 | Present assessments of certainty (or confidence) in the body of evidence for each outcome assessed. | GRADE evidence quality assessment |
| **DISCUSSION** | | |  |
| Discussion | 23a | Provide a general interpretation of the results in the context of other evidence. | Overall Findings |
|  | 23b | Discuss any limitations of the evidence included in the review. | Limitations and future research directions |
|  | 23c | Discuss any limitations of the review processes used. | Overall Findings，Limitations and future research directions |
|  | 23d | Discuss implications of the results for practice, policy, and future research. | Conclusions |
| **OTHER INFORMATION** | | |  |
| Registration and protocol | 24a | Provide registration information for the review, including register name and registration number, or state that the review was not registered. | PROSPERO registration information |
|  | 24b | Indicate where the review protocol can be accessed, or state that a protocol was not prepared. | None |
|  | 24c | Describe and explain any amendments to information provided at registration or in the protocol. | PROSPERO registration information |
| Support | 25 | Describe sources of financial or non-financial support for the review, and the role of the funders or sponsors in the review. | Funding |
| Competing interests | 26 | Declare any competing interests of review authors. | Competing interests |
| Availability of data, code and other materials | 27 | Report which of the following are publicly available and where they can be found: template data collection forms; data extracted from included studies; data used for all analyses; analytic code; any other materials used in the review. | Availability of data and materials |

**S2 Table. Search strategy**

| **Database** | **Search strategy** | |
| --- | --- | --- |
| **PubMed** | **#1** | "osteoarthritis, knee"[MeSH Terms] OR ("osteoarthritis, knee"[MeSH Terms] OR ("osteoarthritis"[All Fields] AND "knee"[All Fields]) OR "knee osteoarthritis"[All Fields] OR ("knee"[All Fields] AND "osteoarthritides"[All Fields]) OR "knee osteoarthritides"[All Fields]) OR ("osteoarthritis, knee"[MeSH Terms] OR ("osteoarthritis"[All Fields] AND "knee"[All Fields]) OR "knee osteoarthritis"[All Fields] OR ("knee"[All Fields] AND "osteoarthritis"[All Fields])) OR ("osteoarthritis, knee"[MeSH Terms] OR ("osteoarthritis"[All Fields] AND "knee"[All Fields]) OR "knee osteoarthritis"[All Fields] OR "osteoarthritis of knee"[All Fields]) OR ("osteoarthritis, knee"[MeSH Terms] OR ("osteoarthritis"[All Fields] AND "knee"[All Fields]) OR "knee osteoarthritis"[All Fields] OR "osteoarthritis of the knee"[All Fields]) |
|  | **#2** | "Ozone"[MeSH Terms] OR (("ozonated"[All Fields] OR "ozonating"[All Fields] OR "ozonation"[All Fields] OR "ozonations"[All Fields] OR "Ozone"[Supplementary Concept] OR "Ozone"[All Fields] OR "Ozone"[MeSH Terms] OR "ozone s"[All Fields] OR "ozonization"[All Fields] OR "ozonized"[All Fields] OR "ozonizer"[All Fields]) AND ("therapeutics"[MeSH Terms] OR "therapeutics"[All Fields] OR "therapies"[All Fields] OR "therapy"[MeSH Subheading] OR "therapy"[All Fields] OR "therapy s"[All Fields] OR "therapys"[All Fields])) OR "Oxygen-Ozone"[All Fields] OR ("O3"[All Fields] AND ("therapeutics"[MeSH Terms] OR "therapeutics"[All Fields] OR "therapies"[All Fields] OR "therapy"[MeSH Subheading] OR "therapy"[All Fields] OR "therapy s"[All Fields] OR "therapys"[All Fields])) |
|  | **#3** | "Randomized Controlled Trials as Topic"[MeSH Terms] OR "randomized controlled trial"[Title/Abstract] OR "clinical trials randomized"[Title/Abstract] OR "trials randomized clinical"[Title/Abstract] OR "controlled clinical trials"[Title/Abstract] OR "controlled clinical trial"[Title/Abstract] OR "Randomized"[Title/Abstract] OR "Randomized"[Title/Abstract] OR "trial"[Title/Abstract] OR "placebo"[Title/Abstract] |
|  | **#1AND #2 AND #3** | |
| **Embase** | **#1** | 'knee osteoarthritis'/exp OR 'arthrosis, knee' OR 'femorotibial arthrosis' OR 'gonarthrosis' OR 'knee arthrosis' OR 'knee joint arthrosis' OR 'knee joint osteoarthritis' OR 'knee osteo-arthritis' OR 'knee osteo-arthrosis' OR 'knee osteoarthrosis' OR 'osteoarthritis, knee' OR 'osteoarthrosis, knee' OR 'knee osteoarthritis' |
|  | **#2** | 'ozone'/exp OR 'oxygen, triplet' OR 'ozon' OR 'triplet oxygen' OR 'ozone' OR 'ozone therapy'/exp OR 'ozone therapy' OR 'oxygen-ozone' OR 'o3 therapy' |
|  | **#3** | 'randomized controlled trial'/exp AND topic OR 'pragmatic clinical trials as topic' OR 'randomized controlled trials as topic' OR 'controlled trial, randomized' OR 'randomised controlled study' OR 'randomised controlled trial' OR 'randomized controlled study' OR 'trial, randomized controlled' OR 'randomized controlled trial' |
|  | **#1AND #2 AND #3** | |
| **Cochrane Library** | **#1** | MeSH descriptor: [Osteoarthritis, Knee] explode all trees |
|  | **#2** | (Knee Osteoarthritides):ti,ab,kw OR (Knee Osteoarthritis):ti,ab,kw OR (Osteoarthritis of Knee):ti,ab,kw OR (Osteoarthritis of the Knee):ti,ab,kw OR (Osteoarthritis, Knee):ti,ab,kw |
|  | **#3** | #1 OR #2 |
|  | **#4** | MeSH descriptor: [Ozone] explode all trees |
|  | **#5** | (Ozone):ti,ab,kw OR (Ozone therapy):ti,ab,kw OR (Oxygen-Ozone):ti,ab,kw OR (O3 therapy):ti,ab,kw |
|  | **#6** | #4 OR #5 |
|  | **#7** | MeSH descriptor: [Randomized Controlled Trials as Topic] explode all trees |
|  | **#8** | (Randomized Controlled Trial):ti,ab,kw OR (Controlled Clinical Trials, Randomized):ti,ab,kw OR (Trials, Randomized Clinical):ti,ab,kw OR (Clinical Trials, Randomized):ti,ab,kw OR (randomized):ti,ab,kw |
|  | **#9** | #7 OR #8 |
|  | **#3AND #6 AND #9** | |
| **Web of science** | **#1** | Osteoarthritis, Knee (All Fields) OR Knee Osteoarthritides (All Fields) OR Knee Osteoarthritis (All Fields) OR Osteoarthritis of Knee (All Fields) OR Osteoarthritis of the Knee (All Fields) |
|  | **#2** | Ozone (All Fields) OR Ozone therapy (All Fields) OR Oxygen-Ozone (All Fields) OR O3 therapy (All Fields) |
|  | **#3** | Randomized Controlled Trials (All Fields) OR randomized controlled trial (All Fields) OR Clinical Trials, Randomized (All Fields) OR Trials, Randomized Clinical (All Fields) OR Controlled Clinical Trials (All Fields) OR controlled clinical trial (All Fields) OR randomized (All Fields) |
|  | **#1AND #2 AND #3** | |
| **Scopus** | **#1** | TITLE-ABS-KEY ( "Osteoarthritis, Knee" OR "Knee Osteoarthritides" OR "Knee Osteoarthritis" OR "Osteoarthritis of Knee" OR "Osteoarthritis of the Knee" ) |
|  | **#2** | TITLE-ABS-KEY ( "ozone" OR "Ozone therapy" OR "Oxygen-Ozone" OR "O3 therapy") |
|  | **#3** | TITLE-ABS-KEY ( "Randomized Controlled Trials" OR "randomized controlled trial" OR "Clinical Trials, Randomized" OR "Trials, Randomized Clinical" OR "Controlled Clinical Trials" OR "controlled clinical trial" OR "randomized" ) |
|  | **#1AND #2 AND #3** | |

**S3 Table. Raw data and calculation of mean and SD for all RCTs**

See Raw data and calculation of mean and SD for all RCTs—Excel

**S4 Table. Excluded studies and reasons**

**Records excluded based on title/abstract，with reasons（n=83）**

-Not RCT（n=50）


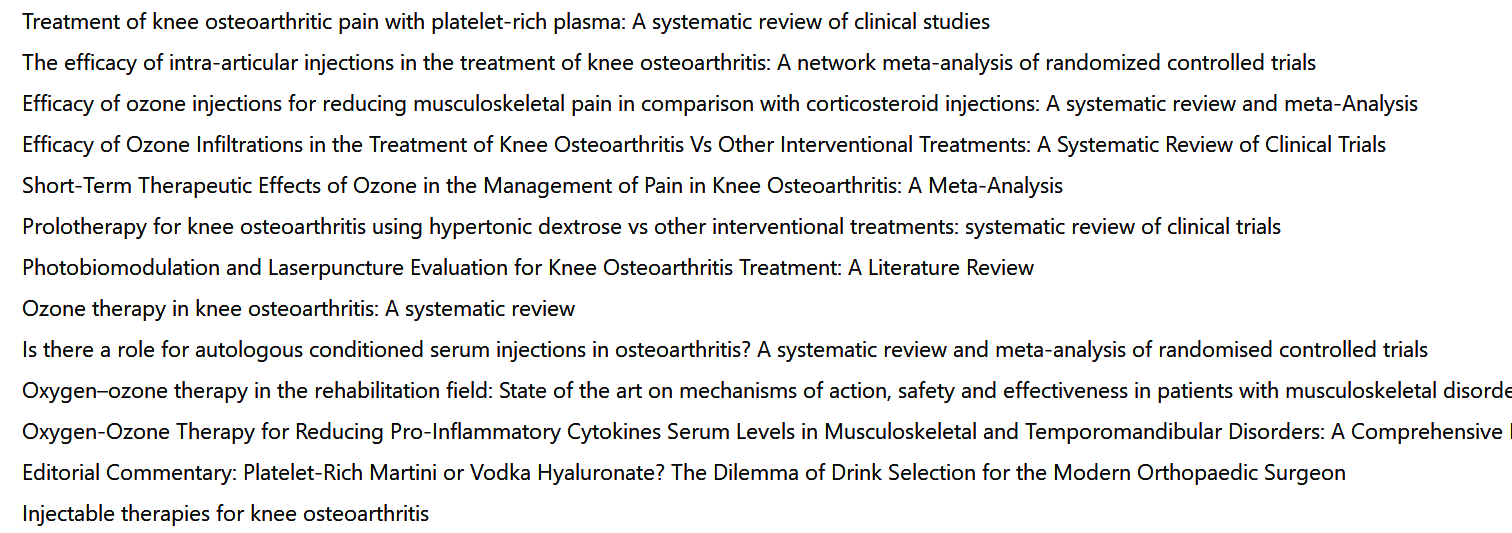

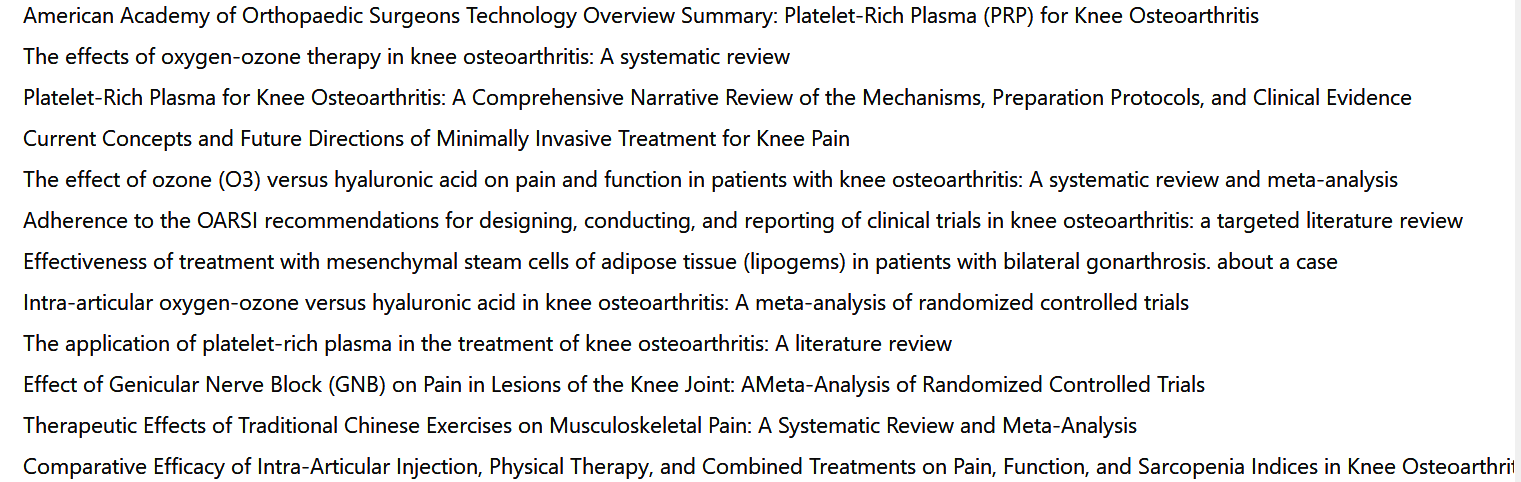

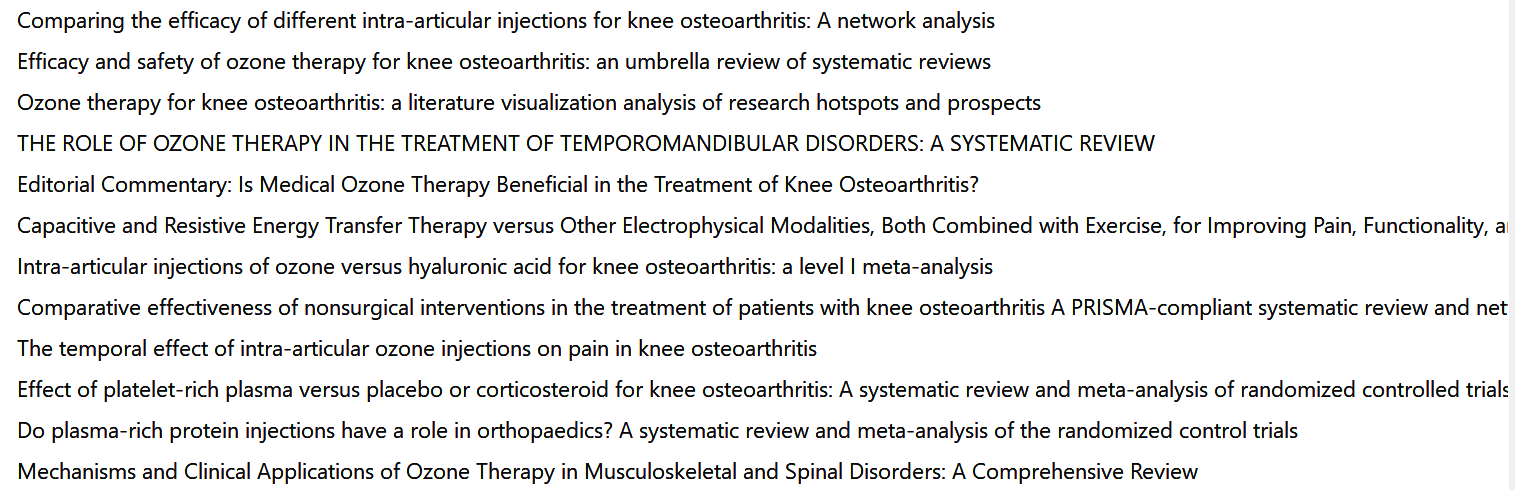

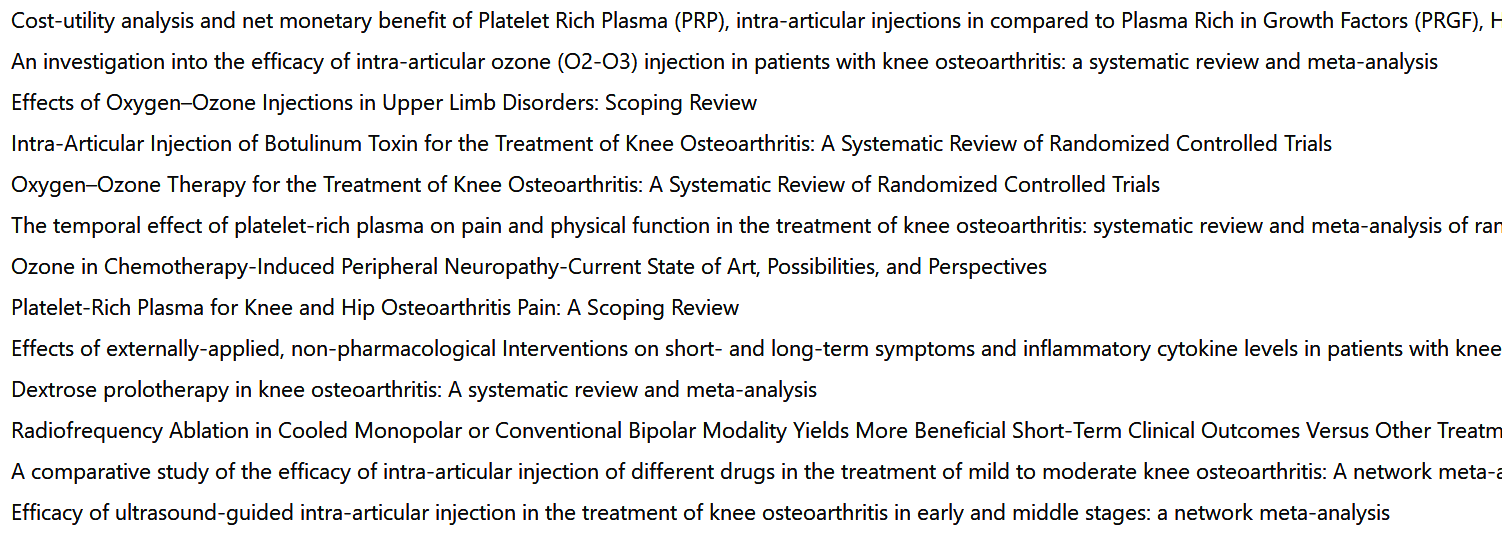


-Not eligible to participates (n=13)


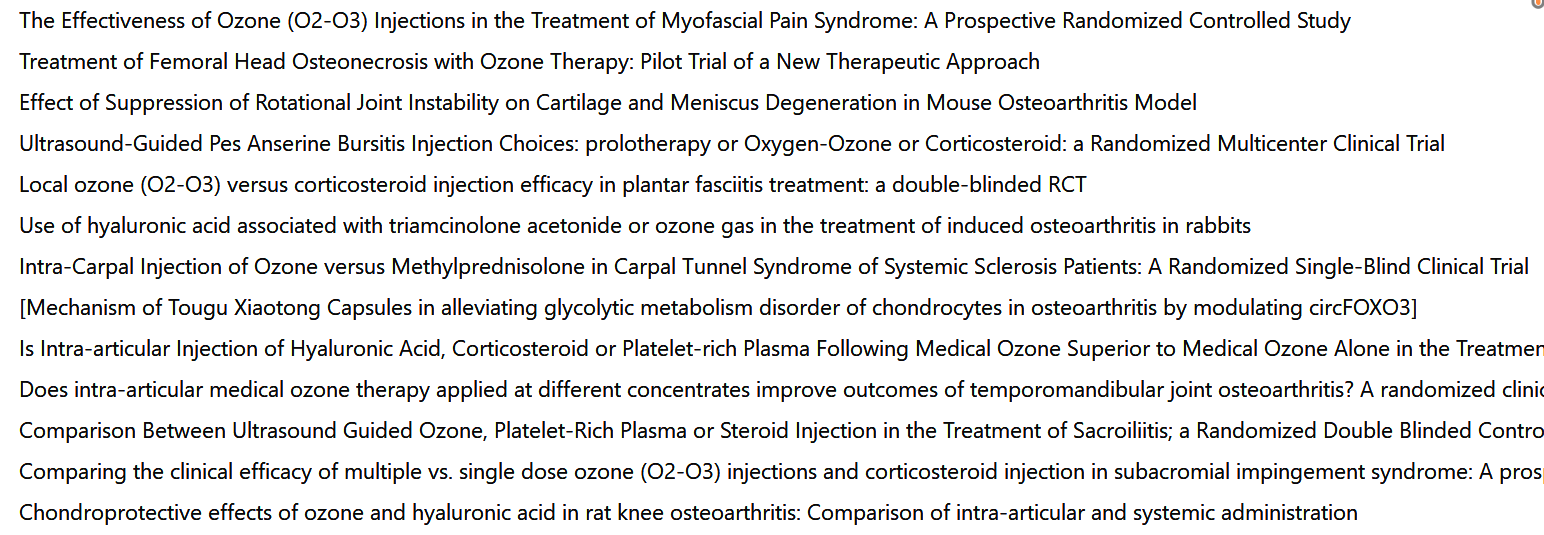


-Not eligible for intervention (n=4)


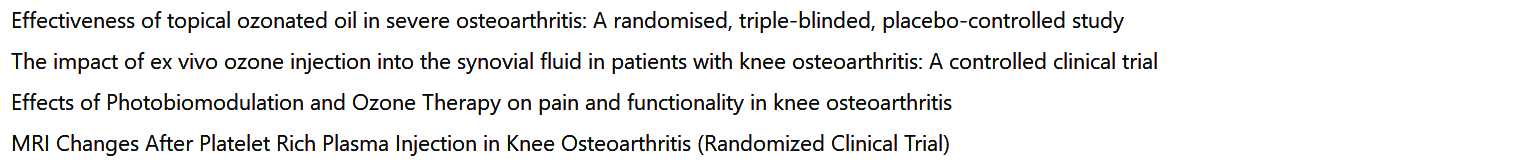


-Combination therapies (n=16)


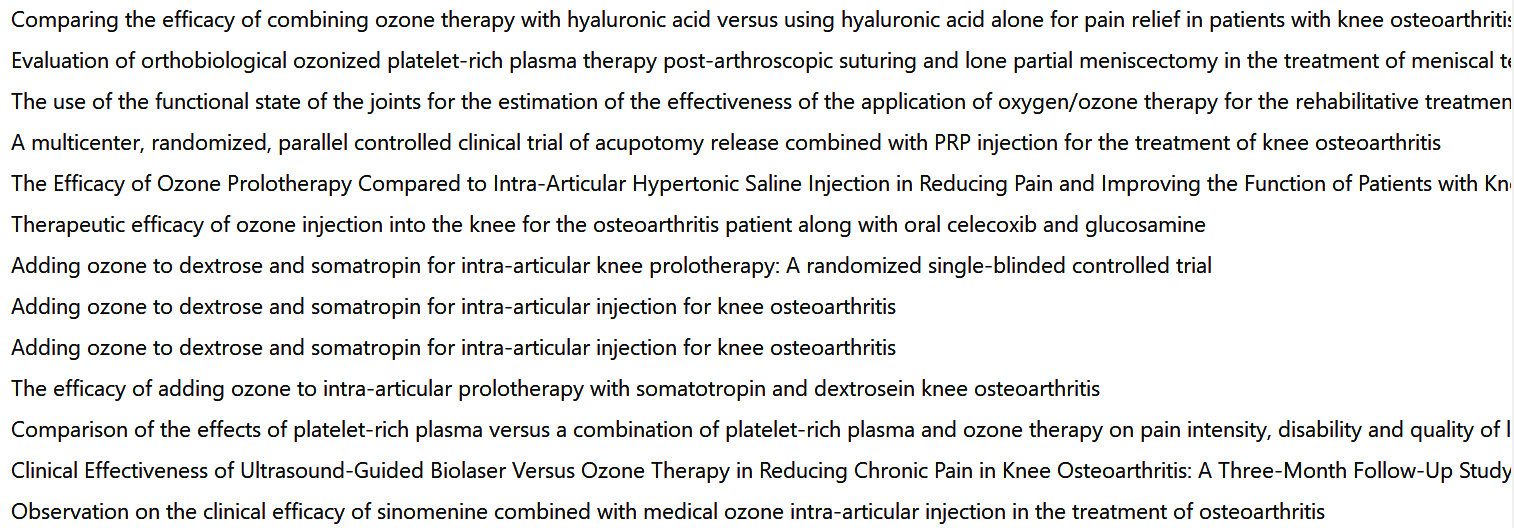

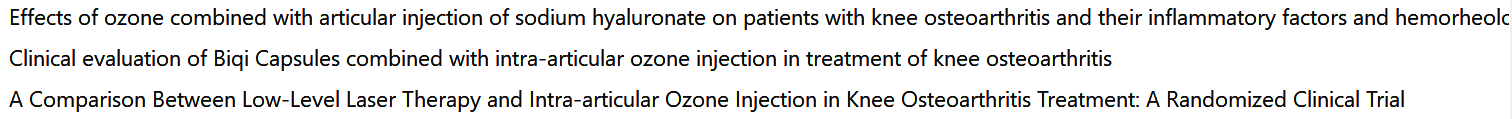


**Full-text articles excluded,with Eligibility reasons(n=19)**

-Not RCT (n=1)


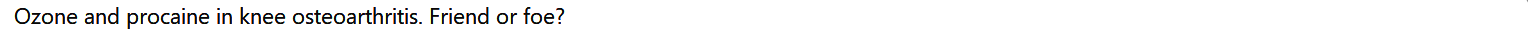


-Not eligible for intervention (n=1)


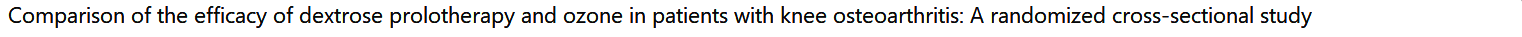


-Not meeting the outcome indicators Eligibility（n=1）


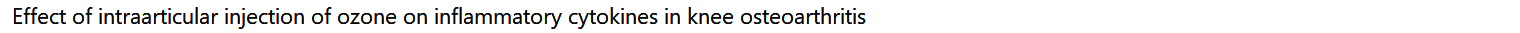


-Non-English and Chinese(n=1)


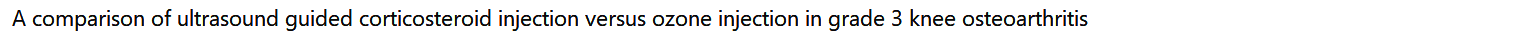


-Data is incomplete (n=1)


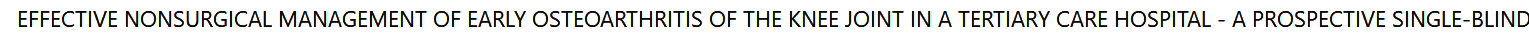


-No raw data (n=3)


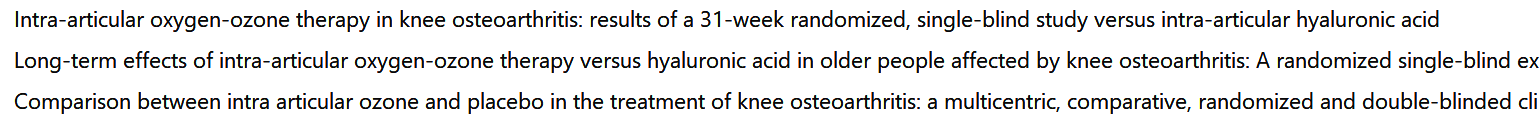


-Full text not found (n=9)


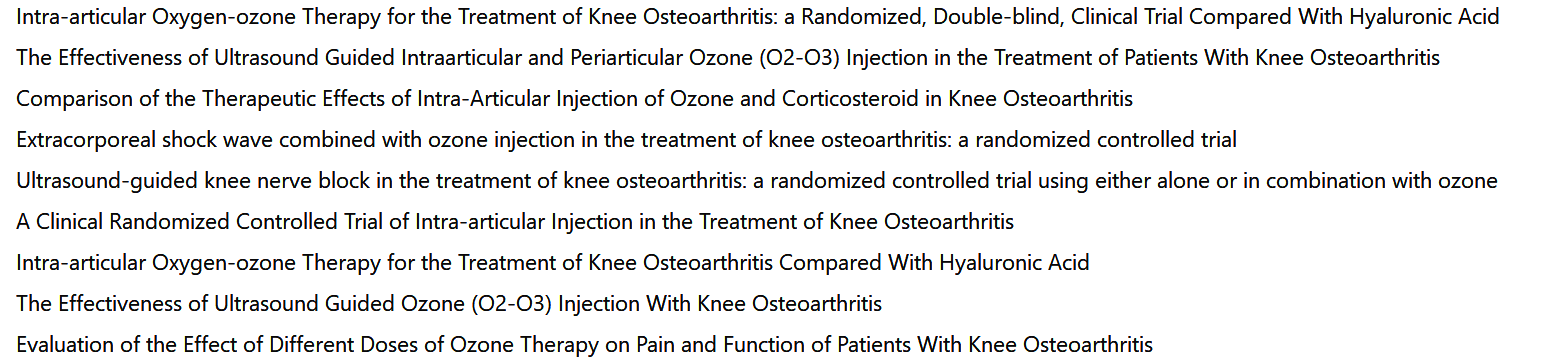


-Duplicate literature (n=2)


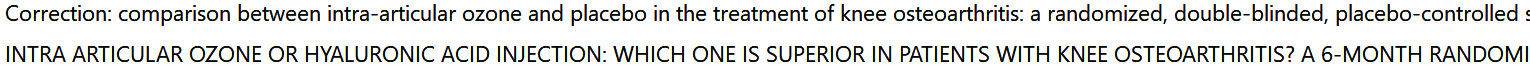


S5 Table. Reasons for risk of bias

| **Reasons for risk of bias** | | | | | | | | | | | | | | | |
| --- | --- | --- | --- | --- | --- | --- | --- | --- | --- | --- | --- | --- | --- | --- | --- |
| **study** | | **Random sequence generation (selection bias)** | | **Allocation concealment (selection bias)** | | **Blinding of participants and personnel (performance bias)** | | **Blinding of outcome assessment (detection bias)** | | **Incomplete outcome data (attrition bias)** | | **Selective reporting (reporting bias)** | | **Other bias** | |
| 1 | A. GIOMBINI  et al.2016 | Low risk of bias | Computer-generated random sequence list | Unclear risk of bias | No specific allocation hiding method mentioned | High risk of bias | Not blinded | Unclear risk of bias | It is not clearly stated whether the outcome assessors were aware of the group assignments. | Low risk of bias | Assume all patients have completed treatment and evaluation | Low risk of bias | Fully reported the preset ending | Unclear risk of bias | The sample size is relatively small, which may affect the generalizability of the results. |
| 2 | Seyed Ahmad Raeissadat et al.2021 | Low risk of bias | Random allocation | Low risk of bias | Use sealed envelopes to ensure the confidentiality of allocation results | Low risk of bias | Not completely blinded, but the outcome is unlikely to be affected by the lack of blinding. | Low risk of bias | The doctors responsible for outcome assessment are unaware of the group assignments, and the subjects complete the questionnaires with the assistance of blinded assessors. | Low risk of bias | Reported the number of lost-to-follow-up cases and the reasons | Low risk of bias | Fully reported the preset ending | Unclear risk of bias | No placebo control group was set, which may affect the results. |
| 3 | Carlos Ce ar Lopes de Jesus  et al.2017 | Low risk of bias | Random allocation | Low risk of bias | Store the randomization scheme in a sealed opaque envelope | Low risk of bias | Blinded to the subjects and primary researchers, and unlikely to be unblinded | Low risk of bias | Blinding of researchers for outcome assessment | Low risk of bias | Reported the number of lost-to-follow-up cases and the reasons | Low risk of bias | Fully reported the preset ending | Low risk of bias | There are no other obvious sources of bias |
| 4 | Zahra Arjmanddoust  et al.2025 | Low risk of bias | Computer-generated random sequence list | Low risk of bias | Store the randomization scheme in a sealed opaque envelope | Low risk of bias | Blinded to the subjects and primary researchers, and unlikely to be unblinded | Low risk of bias | The doctor responsible for outcome assessment is unaware of the group assignments. | Low risk of bias | Reported the number of lost-to-follow-up cases and the reasons | Low risk of bias | Fully reported the preset ending | Unclear risk of bias | The sample size is relatively small, which may affect the generalizability of the results. |
| 5 | Seyed Ahmad Raeissadat et al.2018 | Low risk of bias | Random allocation | Unclear risk of bias | No specific allocation hiding method mentioned | High risk of bias | Intervention implementers are aware of group assignments | Low risk of bias | Blinding of researchers for outcome assessment | Low risk of bias | Reported the number of lost-to-follow-up cases and the reasons | Low risk of bias | Fully reported the preset ending | Low risk of bias | There are no other obvious sources of bias |
| 6 | Cristiano Sconza et al.2025 | Low risk of bias | Random allocation | Low risk of bias | Use sealed envelopes for anonymous distribution | High risk of bias | Intervention implementers may be aware of group assignments | Low risk of bias | Blinding of researchers for outcome assessment | Low risk of bias | Reported the number of lost-to-follow-up cases and the reasons | Low risk of bias | Fully reported the preset ending | Low risk of bias | There are no other obvious sources of bias |
| 7 | Cristiano Sconza et al.2023 | Low risk of bias | Random allocation | Low risk of bias | Keep the random allocation scheme in a sealed envelope | High risk of bias | Intervention implementers may be aware of group assignments | Low risk of bias | Blinding of researchers for outcome assessment | Low risk of bias | Reported the number of lost-to-follow-up cases and the reasons | Low risk of bias | Fully reported the preset ending | Unclear risk of bias | The sample size is relatively small, which may affect the generalizability of the results. |
| 8 | Ghada Abd et al.2025 | Low risk of bias | Random allocation | Low risk of bias | Keep the random allocation scheme in a sealed envelope | Unclear risk of bias | Not clearly stated | Unclear risk of bias | It is not clearly stated whether the outcome assessors were aware of the group assignments. | Low risk of bias | Assume all patients have completed treatment and evaluation | Low risk of bias | Fully reported the preset ending | Unclear risk of bias | The sample size is relatively small, which may affect the generalizability of the results. |
| 9 | Arash Babaei-Ghazani et al.2018 | Low risk of bias | Random allocation | Low risk of bias | Store the randomization scheme in a sealed opaque envelope | High risk of bias | No blinding was applied to the intervention implementers | Low risk of bias | Blinding of researchers for outcome assessment | Low risk of bias | All patients completed treatment and evaluation | Low risk of bias | Fully reported the preset ending | Unclear risk of bias | The sample size is relatively small, which may affect the generalizability of the results. |
| 10 | Nahla M. Gaballa et al.2018 | Low risk of bias | Random allocation | Unclear risk of bias | No specific allocation hiding method mentioned | Unclear risk of bias | Not clearly stated | Unclear risk of bias | It is not clearly stated whether the outcome assessors were aware of the group assignments. | Low risk of bias | Assume all patients have completed treatment and evaluation | Low risk of bias | Fully reported the preset ending | Unclear risk of bias | The sample size is relatively small, which may affect the generalizability of the results. |
| 11 | Tahir Mutlu Duymus et al.2017 | Low risk of bias | Random allocation | Unclear risk of bias | No specific allocation hiding method mentioned | Unclear risk of bias | Not clearly stated | Low risk of bias | Results assessed by an independent evaluator | Low risk of bias | Reported the number of people lost to follow-up | Low risk of bias | Fully reported the preset ending | Unclear risk of bias | The sample size is relatively small, which may affect the generalizability of the results. |
| 12 | Masoud Hashemi et al.2015 | Low risk of bias | Random allocation | Unclear risk of bias | No specific allocation hiding method mentioned | Unclear risk of bias | Not clearly stated | Unclear risk of bias | It is not clearly stated whether the outcome assessors were aware of the group assignments. | Low risk of bias | Assume all patients have completed treatment and evaluation | Low risk of bias | Fully reported the preset ending | Unclear risk of bias | The sample size is relatively small, which may affect the generalizability of the results. |
| 13 | Mahshid Nazarieh et al.2024 | Low risk of bias | Random allocation | Unclear risk of bias | No specific allocation hiding method mentioned | Unclear risk of bias | Not clearly stated | Low risk of bias | The personnel responsible for outcome assessment are not aware of the group assignments. | Low risk of bias | Reported the number of people lost to follow-up | Low risk of bias | Fully reported the preset ending | Unclear risk of bias | The sample size is relatively small, which may affect the generalizability of the results. |
| 14 | Li JuanHong et al.2021 | Low risk of bias | Random Number Table Method | Unclear risk of bias | No specific allocation hiding method mentioned | Unclear risk of bias | Not clearly stated | Unclear risk of bias | It is not clearly stated whether the outcome assessors were aware of the group assignments. | Low risk of bias | Reported the number of lost-to-follow-up cases and the reasons | Low risk of bias | Fully reported the preset ending | Unclear risk of bias | There is no sufficient reason to suggest that the existing issues will introduce bias. |
| 15 | Sefa Gümrük Aslan et al.2024 | Low risk of bias | Random allocation | Unclear risk of bias | No specific allocation hiding method mentioned | High risk of bias | No blinding was applied to the participants or the implementers | Low risk of bias | Blinding of researchers assessing the outcomes | Low risk of bias | Reported the number of lost-to-follow-up cases and the reasons | Low risk of bias | Fully reported the preset ending | Unclear risk of bias | There is no sufficient reason to suggest that the existing issues will introduce bias. |
| 16 | Fernandez-Cuadros et al.2019 | Low risk of bias | Random Number Table Method | Unclear risk of bias | No specific allocation hiding method mentioned | Unclear risk of bias | Not clearly stated | Unclear risk of bias | It is not clearly stated whether the outcome assessors were aware of the group assignments. | Low risk of bias | Reported the number of lost-to-follow-up cases and the reasons | Low risk of bias | Fully reported the preset ending | Unclear risk of bias | The sample size is relatively small, which may affect the generalizability of the results. |

**S1 Figure.Analysis of Heterogeneity Causes**

**WOMAC Total T1**

**
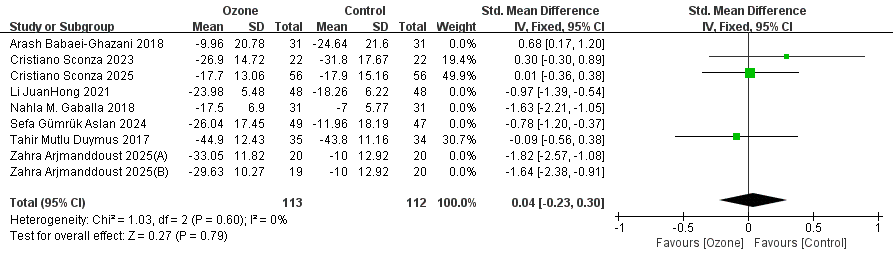
**

**WOMAC Pain T2**


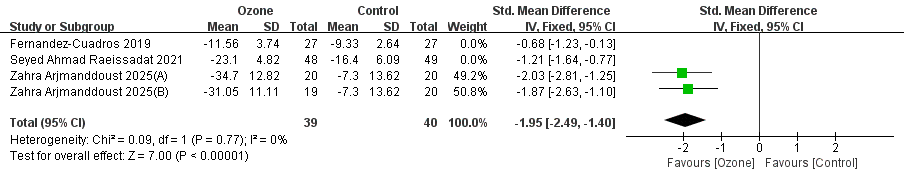


**WOMAC Function T2**


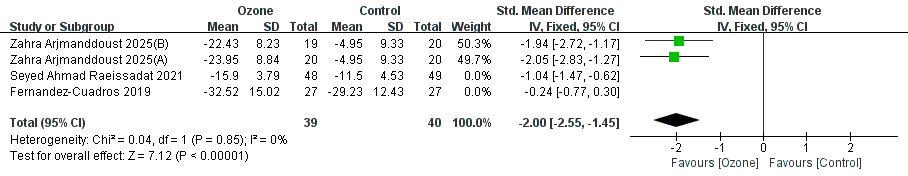


**WOMAC Stiffness T2**


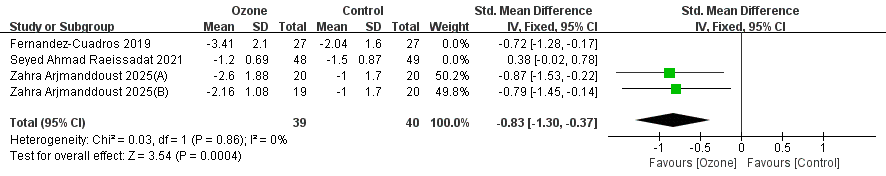


**WOMAC Total T3**


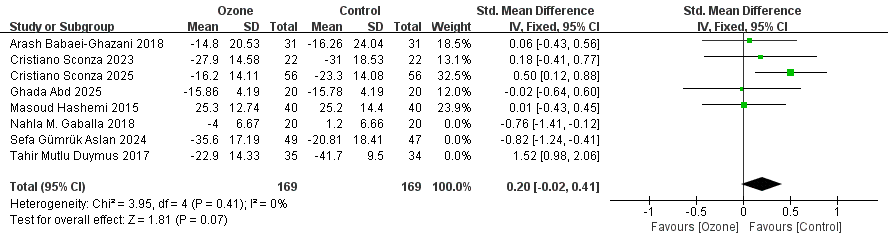


**WOMAC Total T6**


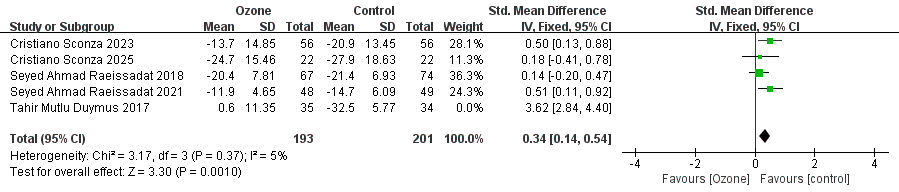


**WOMAC Total T12**


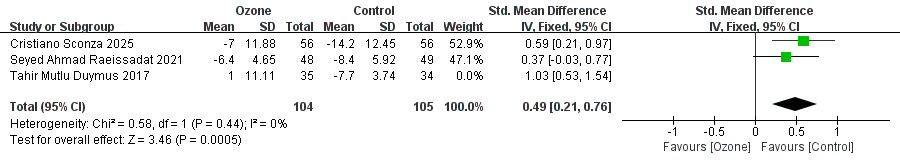


**S2 Figure. Sensitivity Analysis**

**WOMAC Total T1**

**WOMAC Pain T2**

**WOMAC Stiffness T2**

**WOMAC Function T2**

**WOMAC Total T3**

**WOMAC Total T6**

**WOMAC Total T12**

**VAS T1**

**VAS T2**

**VAS T3**

**VAS T6**

**VAS T12**

**S3 Figure. Subgroup analyses of WOMAC and VAS outcomes stratified by control type (placebo/oxygen vs. hyaluronic acid) at different follow-up time points**

**VAS：Ozone VS HA**

**
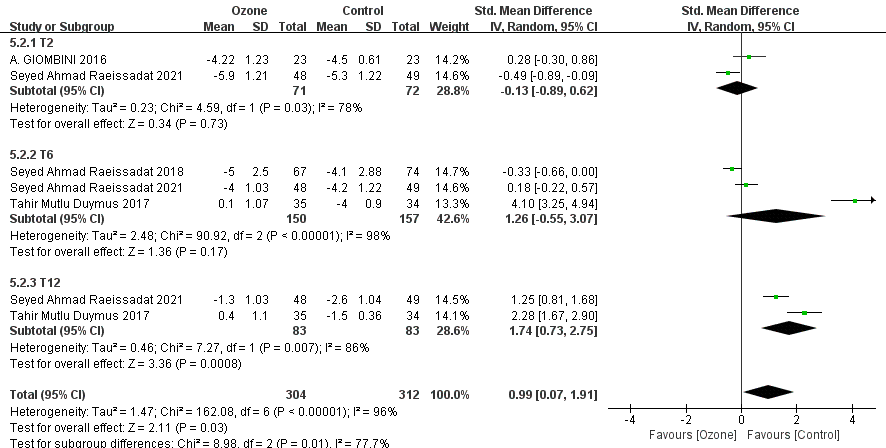
**

**WOMAC：Ozone VS Placebo**

**
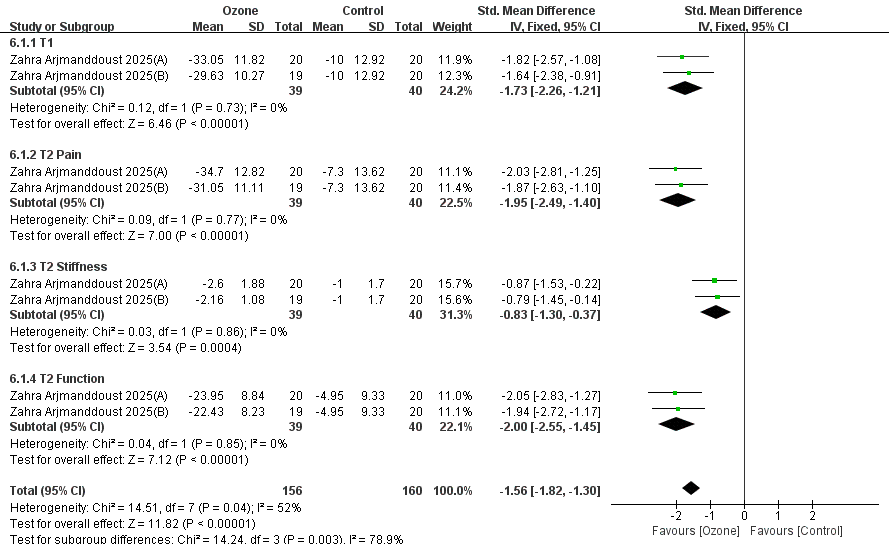
**

**VAS：Ozone VS Placebo**

**
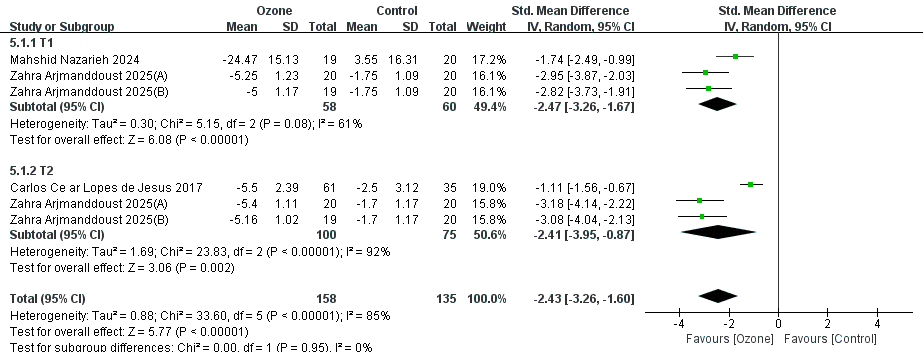
**

**WOMAC：Ozone VS HA**

**
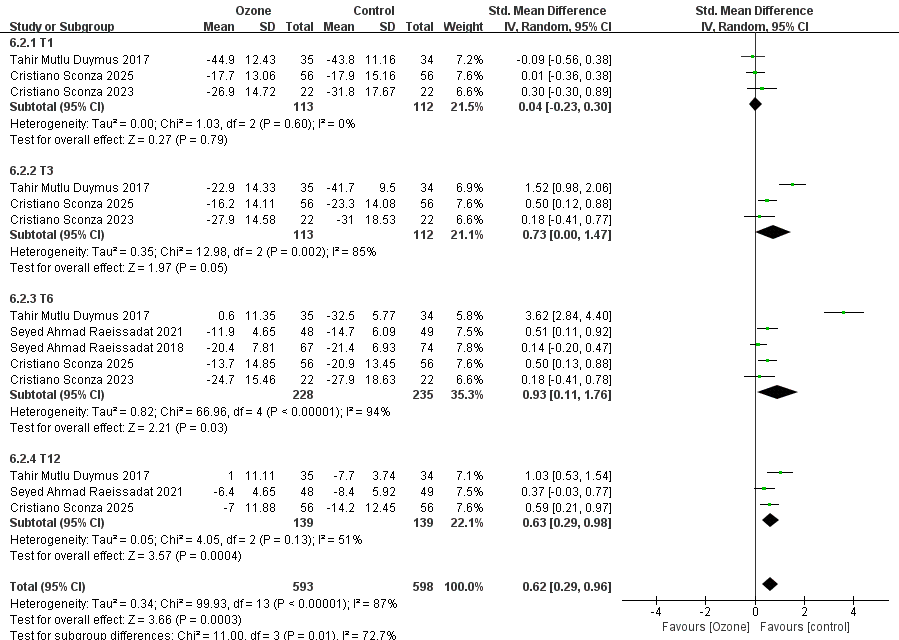
**
